# Analysis of drug-resistant tuberculosis transmission dynamics in China using fractional stochastic model

**DOI:** 10.1371/journal.pone.0335889

**Published:** 2025-11-13

**Authors:** Shaoping Jiang, Hongyan Wang, Yudie Hu

**Affiliations:** School of Mathematics and Computer Science, Yunnan Minzu University, Kunming, Yunnan, China; Recep Tayyip Erdogan Universitesi, TÜRKIYE

## Abstract

This study investigates the dynamics of the drug-resistant tuberculosis model through a fractional stochastic modeling framework. The model employs fractional-order derivatives to capture the memory effects in disease transmission, while Brownian motion is introduced to represent the random disturbances, thereby providing a more realistic description of the disease dynamics. First, a fractional deterministic model based on the Atangana-Baleanu-Caputo derivative was developed, and its optimal parameter values were obtained from the actual data from the case of drug-resistant tuberculosis in China. Second, the existence and uniqueness of the solution of the fractional stochastic model were proved, and its numerical solution was explored. Furthermore, the impacts of different interventions strategies on the control of drug-resistant tuberculosis in China were compared. The results demonstrate that the combined interventions exhibit superior efficacy compared to any single intervention. Numerical simulations of deterministic and fractional stochastic models verify the effects of memory and random effects on drug-resistant tuberculosis. It was found that as the noise level increases, the degree of random perturbation in the model solution also increases, and higher noise levels may lead to the early disappearance of drug-resistant tuberculosis.

## Introduction

Tuberculosis(TB) is an infectious disease resulting from the bacillus Mycobacterium tuberculosis, which is spread through the air when an infected individual coughs or sneezes. In 2022, tuberculosis became the second most significant cause of death worldwide from a single infectious disease, trailing behind COVID-19 [1]. Drug-resistant tuberculosis is caused by already resistant strains of Mycobacterium tuberculosis or incomplete treatment of sensitive strains. Compared with the treatment of Drug-sensitive tuberculosis(DS-TB), the treatment of Drug-resistant tuberculosis(DR-TB) is costly, time-consuming and less effective [2]. DR-TB continues to pose a public health threat [1].

Mathematical models have been extensively employed to study the dynamics of infectious diseases, offering effective discernment regarding the trends of their transmission. In 1962, Waaler et al. used mathematical modeling for the first time to reveal the epidemiological characteristics of tuberculosis [3]. Since then, many scholars have used the model as a basis to construct new integer-order mathematical models to explore the progression of TB [4–7]. Compared with traditional models of integer order, fractional order models can capture memory and hereditary characteristics more accurately [8]. In recent years, numerous authors have made significant contributions to the modeling of disease dynamics by applying fractional calculus to illustrate memory effects in disease transmission [9–11]. Kubra [12] et al. used Atangana-Baleanu fractal-fractional derivatives to construct a model for studying monkeypox outbreaks and conducted qualitative analysis on the model. The proposed model fitted the actual data of USA well. Rahman [13] explored a fractional order mathematical model for the incomplete treatment of TB and utilized the fractional Adam Bashforth technique to numerically solve the model. Omame et al. [14] studied and analyzed a fractional order model of co-infection of COVID-19 and TB. The numerical results showed that reducing the risk of COVID-19 infection among individuals with latent TB infection can effectively reduce the co-infection of the two diseases. Padder et al. [15] developed a tumor model by employing the Caputo fractional order derivative and investigated the dynamical behavior of the model. This Caputo fractional order derivative model provides greater accuracy compared to the classical integer-order derivative.

The study of stochastic infectious disease dynamics models has evolved into a systematic theoretical framework both domestically and internationally, which incorporates stochastic processes to quantify the impact of uncertainty factors on disease transmission. Anwarud Din employed a delayed stochastic epidemic model incorporating general incidence rates, time-delayed transmission, and the concept of cross-immunity to investigate the spread of infectious diseases [16]. Anwarud Din et al. established a stochastic HBV model by introducing Gaussian white noise and time-delay factors, and obtained the conditions for virus extinction and the existence of a stationary distribution in the model [17]. Khan et al. employed an innovative fractional-order mathematical model integrating fractional calculus and differential equations to investigate the pathogenesis of food allergies in populations using antacid medications, and analyzed the complex dynamics of food allergy epidemics along with their interactions with antacid usage [18]. Tul Ain et al. [19] developed a cholera model incorporating stochastic differential equations based on the transmission characteristics of cholera, through analyzing the mechanism of disease onset and symptom manifestation following individual exposure to pathogen concentrations. Authors Ucar et al. [20] developed a novel model to analyze the interactions between tumor cells and macrophages. By incorporating stochastic factors, the model accounts for inherent random fluctuations within the system and provides in-depth insights into the potential mechanisms through which stochastic elements may influence tumor progression and immune response. Recently, Andrawus et al. [21] developed a compartmental mathematical model that describes the transmission dynamics of the monkeypox virus. The results demonstrate that contact tracing, disease surveillance isolation, and vaccination can completely prevent human-to-human transmission of monkeypox. Based on the Caputo fractional-order derivative, Authors Naik et al. [22] investigated the dynamic characteristics of a co-infection model of Human Immunodeficiency Virus (HIV) and Hepatitis C Virus (HCV). The study provides optimal treatment strategies and offers theoretical support for understanding the complex interactions and co-evolutionary dynamics of HIV/HCV.

Given the numerous uncertainties in the disease transmission process, the classical fractional order derivative is generally not an adequate explanation for its stochastic nature. Mathematicians then introduced the concept of Brownian motion to capture randomness [23]. Researchers have developed fractional stochastic models to describe the spread of disease [24–27]. For instance, Bonyah et al. [28] studied the dynamics of monkeypox by exploying a fractional stochastic model and verified the impact of fractional order derivatives on the dynamics of monkeypox. Chukwu [29] developed a fractional order stochastic model for TB. A comparison among the three operators revealed that the Caputo-Fabrizio operator exhibits greater randomness than both the Atangana-Baleanu operator and the Caputo operator. Mangal et al. [30] revealed the role of public awareness on the outbreaks through fractional stochastic modelling. Rashid et al. [31] developed a fractional stochastic model of measles that incorporates dual medication immunization and presented numerical simulation results for different fractional orders and stochastic strengths. However, as far as we know, none of the existing fractional order models have investigated the stochastic nature of the DR-TB.

In 2014, the World Health Organization (WHO) developed the End TB Strategy, which aims to reduce the incidence of TB by 90 percent by 2035 compared to 2015 [32]. Taking Chinese DR-TB patients as research subjects, we established a fractional stochastic DR-TB model by considering memory effects and randomness into account in the process of DR-TB transmission on the basis of existing models.

The paper is organized as follows: Section 0 introduces the basic mathematics about fractional order calculus equations. Section 0 provides a detailed description of the model. Section analyzes the dynamics of the model. In Section 1, the existence and uniqueness of the solution to the model are explored. Section 2 discusses the numerical solution of the fractional stochastic model. Numerical simulations are performed, and the results are discussed in Section . Finally, Section summarizes the main results of the study.

## Mathematical preliminaries

**Definition 1.**

[33] Let $h\in H^{1}(a,b),b>a,q\in [0,1]$, the definition of the new fractional derivative is given as:


$${\;}_{0}^{ABC}D_{t}^{q}h(t)=\frac{ABC(q)}{1-q}\int_{a}^{t}E_{q}\left[\frac{-q}{1-q}(t-x{)}^{q}\right]\frac{d}{dx}h(x)dx,$$

where, ${\;}_{0}^{ABC}D_{t}^{q}$ is fractional operator with Mittage-Leffler kernel in the Caputo sense with order q with respect to t and ABC(q) is a normalization function. And $ABC(q)=1-q+\frac{q}{\Gamma (1-q)}$.

**Definition 2.**

[33] The Mittag-Leffler function is defined as


$$E_{\alpha ,\beta }(z)=\sum_{k=0}^{\infty }\frac{z^{k}}{\Gamma (k\alpha +\beta )},$$

where, α,β>0 and $E_{\alpha ,1}(z)=E_{\alpha }(z)$.

**Lemma 1.**

[34] The connection between Atangana-Baleanu-Caputo operator and the Laplace transform


$$L{[}_{0}^{ABC}D_{t}^{q}h(t)](s)=\frac{ABC(q)}{1-q}\frac{s^{q}L[h(t)](s)-s^{q-1}h(0)}{s^{q}+\frac{q}{1-q}}.$$

## System description

In this section, we present the formulation of fractional stochastic mathematical model for DR-TB. We will incorporate the stochastic factors in the DR-TB transmission process into the model. In our model, the human population *N*(*t*) is sub-divided into seven classes: the susceptible individuals(*S*), the DS-TB latent individuals (*E*_*s*_), the DS-TB individuals (*I*_*s*_), the DS-TB recovered individuals (*R*_*s*_), the DR-TB latent individuals (*E*_*r*_), the DR-TB individuals (*I*_*r*_), the DR-TB recovered individuals (*R*_*r*_). Furthermore, $N(t)=S(t)+E_{s}(t)+I_{s}(t)+R_{s}(t)+E_{r}(t)+I_{r}(t)+R_{r}(t)$.

The Atangana-Baleanu-Caputo operator is capable of simultaneously describing the memory effects and non-local characteristics of a system. The fractional order q∈(0,1) characterizes the temporal memory properties in the process of disease transmission. When q→1, the system exhibits standard dynamic behavior, whereas as q decreases, the infection risk among susceptible individuals is influenced by historical exposure levels over a longer duration. This aligns more closely with the biological features of tuberculosis, which has a long incubation period and time-cumulative effects. The non-local transmission characteristic of this operator, unlike the local transmission described by traditional integer-order derivatives, allows it to better represent non-contact transmission mechanisms such as those facilitated by social networks and spatial mobility. This highly corresponds to the nature of Mycobacterium tuberculosis transmission via long-distance aerosol spread.

Our model is based on the model established by Xu [35]. In Xu’s article, the relapse rates of DS-TB and DR-TB are low. Thus, for convenience, we do not consider the relapse rates of DS-TB and DR-TB separately in our model. Susceptible individuals become infected with TB by having close contact with *I*_*s*_ and *I*_*r*_ at contact rates of $\beta_{s}$ and $\beta_{r}$, respectively, and they become the classes *E*_*s*_ and *E*_*r*_. Individuals in the class *E*_*s*_ and *E*_*r*_ transfer to the class *I*_*s*_ and *I*_*r*_ at a rate of *v*. *I*_*s*_ may recover to become *R*_*s*_ at the rate of *rc*_*s*_ during the course of treatment, or they may acquire resistance at the rate of (1–*r*)*c* due to other reasons such as non-standard treatment of DS-TB patients, allowing *I*_*s*_ to become *I*_*r*_. At the same time, *I*_*s*_ and *I*_*r*_ may die from the disease with a mortality rate of *μ*. *I*_*r*_ changes to *R*_*r*_ at a cure rate of *c*_*r*_ during the course of treatment. Additionally, it is considered that each population group has a natural death rate of $\mu^{\prime }$. Assuming that the number of DR-TB patients per year is 30 percent of the total number of TB patients [35].

**Fig 1:**
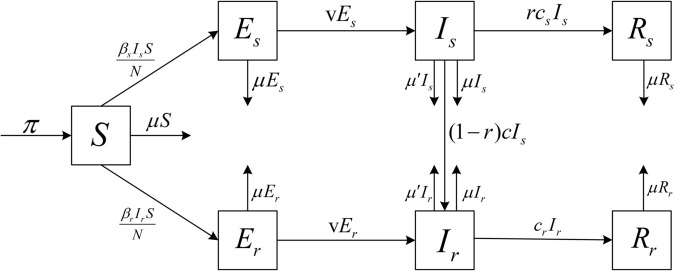
Schematic diagram of transmission pathways for drug-sensitive (DS) and drug-resistant (DR) tuberculosis.

The biological meanings of the parameters are given in Table 1, and the transfer diagram of DS-TB and DR-TB is illustrated in Fig 1. Therefore, we give a model represented by the following dynamical system:

**Table 1:** Parameters and biological meanings.

Parameters	Biological meanings
*δ*	Rate at which susceptible individuals become exposed individuals
$\beta_{s}$	DS-TB transmission rate
$\beta_{r}$	DR-TB transmission rate
*v*	Progression rate
*c*	DS-TB to DR-TB conversion rate
*c*_*s*_	Cure rate of DS-TB
*c*_*r*_	Cure rate of DR-TB
*π*	National births
*r*	Proportion of DS-TB cured cases
*μ*	Natural mortality rate
$\mu^{\prime }$	Mortality rate due to TB

$${\;}_{0}^{ABC}D_{t}^{q}S=\pi -\mu S-\frac{\beta_{s}I_{s}}{N}S-\frac{\beta_{r}I_{r}}{N}S,\;{\;}_{0}^{ABC}D_{t}^{q}E_{s}=\frac{\beta_{s}I_{s}}{N}S-vE_{s}-\mu E_{s},\;{\;}_{0}^{ABC}D_{t}^{q}I_{s}=vE_{s}-[rc_{s}+(1-r)c]I_{s}-(\mu +\mu^{{\;}^{\prime }})I_{s},\;{\;}_{0}^{ABC}D_{t}^{q}R_{s}=rc_{s}I_{s}-\mu R_{s},\;{\;}_{0}^{ABC}D_{t}^{q}E_{r}=\frac{\beta_{r}I_{r}}{N}S-vE_{r}-\mu E_{r},\;{\;}_{0}^{ABC}D_{t}^{q}I_{r}=vE_{r}+(1-r)cI_{s}-(c_{r}+\mu +\mu^{{\;}^{\prime }})I_{r},\;{\;}_{0}^{ABC}D_{t}^{q}R_{r}=c_{r}I_{r}-\mu R_{r}.$$ (1)

Where the initial conditions satisfy the following non-negative conditions: $S(0)\geq 0,E_{s}(0)\geq 0,I_{s}(0)\geq 0,R_{s}(0)\geq 0,E_{r}(0)\geq 0,I_{r}\geq 0,R_{r}\geq 0.$

## Analysis of the system

### 1. Non-negativity of the solution

**Theorem 1.**

The set $\Upsilon =\left\{(S,E_{s},I_{s},R_{s},E_{r},I_{r},R_{r})\in {\mathbb{R}}_{+}^{7}:S+E_{s}+I_{s}+R_{s}+E_{r}+I_{r}+R_{r}\leq \frac{\pi }{\mu }\right\}$ is positivity invariant with respect to the model (1).

**Proof.**

Adding the equations in model (1), we get


$${\;}_{0}^{ABC}D_{t}^{q}(S+E_{s}+I_{s}+R_{s}+E_{r}+I_{r}+R_{r})=\pi -\mu N-\mu^{{\;}^{\prime }}(I_{s}+I_{r}),$$

$${\;}_{0}^{ABC}D_{t}^{q}N\leq \pi -\mu N.$$ (2)

By applying the Laplace transform on the both sides of Eq (2), we obtain,


$$\frac{AB(q)}{1-q}\cdot \frac{s^{q}L\left\{N(t)\right\}-s^{q-1}N(0)}{s^{q}+\frac{q}{1-q}}\leq \frac{\pi }{s}-\mu L\left\{N(t)\right\},$$

$$L\left\{N(t)\right\}\leq \left[\frac{AB(q)s^{q-1}N(0)}{(1-q)s^{q}+q}+\frac{\pi }{s}\right]\cdot \frac{(1-q)s^{q}+q}{AB(q)s^{q}+\mu [(1-q)s^{q}+q]}.$$ (3)

Taking the inverse Laplace Transform on both sides of Eq (3), we obtain,


$$N(t)\leq L^{-1}\left\{\frac{AB(q)s^{q-1}N(0)}{AB(q)s^{q}+\mu [(1-q)s^{q}+q]}+\frac{\pi }{s}\cdot \frac{(1-q)s^{q}+q}{AB(q)s^{q}+\mu [(1-q)s^{q}+q]}\right\},\;=\frac{AB(q)N(0)+\pi (1-q)}{AB(q)+(1-q)\mu }E_{q,1}(-\xi_{1}t^{q})+\frac{\pi q}{AB(q)+(1-q)\mu }E_{q,q+1}(-\xi_{1}t^{q}),$$

where, $\xi_{1}=\frac{q\mu }{AB(q)+(1-q)\mu }$. By the asymptotic behavior of Mittag-Leffler function *E*_*m*,*n*_, we get $N(t)\leq \frac{\pi }{\mu }$, as t→∞. Hence, the region Υ is the positivity invariant region for the model (1).

### 2. Equilibrium points and basic reproduction number

For the equilibrium points of the model, set all the right-hand side equations of model (1) equal to zero. The equilibrium points of the model established in model (1) can be obtained by solving the following system of equations:


$$0=\pi -\mu S-\frac{\beta_{s}I_{s}}{N}S-\frac{\beta_{r}I_{r}}{N}S,\;0=\frac{\beta_{s}I_{s}}{N}S-vE_{s}-\mu E_{s},\;0=vE_{s}-[rc_{s}+(1-r)c]I_{s}-(\mu +\mu^{{\;}^{\prime }})I_{s},\;0=rc_{s}I_{s}-\mu R_{s},\;0=\frac{\beta_{r}I_{r}}{N}S-vE_{r}-\mu E_{r},\;0=vE_{r}+(1-r)cI_{s}-(c_{r}+\mu +\mu^{{\;}^{\prime }})I_{r},\;0=c_{r}I_{r}-\mu R_{r}.$$

According to the definition of the disease-free equilibrium point,all infection compartments are empty, $E_{s}=I_{s}=R_{s}=E_{r}=I_{r}=R_{r}=0$, at this point, $S=\frac{\mu }{\pi }$. We could obtain that the disease-free equilibrium point of model (1) is given by


$$P_{0}^{*}(\frac{\pi }{\mu },0,0,0,0,0,0).$$

To study the disease equilibrium points, we calculate the basic reproduction number(*R*_0_) using the next generation approach method proposed by Van den Driessche and Watmough [36]. Let $x=(E_{s},I_{s},E_{r},I_{r})$, model (1) could be written as


$${\;}_{0}^{ABC}D_{t}^{q}(x)=ℱ-𝒱,$$

where,


$$ℱ=(\begin{array}{c} \frac{\beta_{s}I_{s}S}{N}\\ 0\\ \frac{\beta_{r}I_{r}S}{N}\\ 0 \end{array}),𝒱=(\begin{array}{c} (v+\mu )E_{s}\\ -vE_{s}+[rc_{s}+(1-r)c+\mu +\mu^{\prime }]I_{s}\\ (v+\mu )E_{r}\\ -vE_{r}-(1-r)cI_{s}+(c_{r}+\mu +\mu^{\prime })I_{r} \end{array}),$$

then, take the derivative of ℱ and 𝒱 for x at the disease-free equilibrium point $P_{1}^{*}$, clearly, we can obtain


$$F=\frac{\partial {ℱ}_{i}}{\partial x_{j}}{|}_{P_{0}^{*}}=(\begin{array}{cccc} 0 & \beta_{s} & 0 & 0\\ 0 & 0 & 0 & 0\\ 0 & 0 & 0 & \beta_{r}\\ 0 & 0 & 0 & 0 \end{array}),\;[6pt]V=\frac{\partial {𝒱}_{i}}{\partial x_{j}}{|}_{P_{0}^{*}}=(\begin{array}{cccc} v+\mu & 0 & 0 & 0\\ -v & rc_{s}+(1-r)c+\mu +\mu^{\prime } & 0 & 0\\ 0 & 0 & v+\mu & 0\\ 0 & -(1-r)c & -v & c_{r}+\mu +\mu^{\prime } \end{array}).$$

Thus, the basic reproduction number is defined as $R_{0}=max\left\{R_{0}^{s},R_{0}^{r}\right\}$, where


$$R_{0}^{s}=\frac{\beta_{s}v}{\left(v+\mu )(c+\mu +\mu^{\prime }-cr+c_{s}r\right)},R_{0}^{r}=\frac{\beta_{r}v}{\left(v+\mu )(c_{r}+\mu +\mu^{\prime }\right)}.$$

### 3. The stability at the disease-free equilibrium points

**Theorem 2.**

If $\left|arg(s_{F-V})\right|>\frac{q\pi }{2}$, model (1) is locally asymptotically stable at the disease-free equilibrium $P_{0}^{*}$.

**Proof.**

The Jacobain matrix of model (1) at the disease-free equilibrium $P_{0}^{*}$ is


$${𝕁}_{p_{0}^{*}}=(\begin{array}{ccccccc} -a_{1} & \beta_{s} & 0 & 0 & 0 & 0 & 0\\ v & -a_{2} & 0 & 0 & 0 & 0 & 0\\ 0 & 0 & -a_{1} & \beta_{r} & 0 & 0 & 0\\ 0 & (1-r)c & v & -a_{3} & 0 & 0 & 0\\ 0 & -\beta_{s} & 0 & -\beta_{r} & -\mu & 0 & 0\\ 0 & rc_{s} & 0 & 0 & 0 & -\mu & 0\\ 0 & 0 & 0 & c_{r} & 0 & 0 & -\mu \end{array}),$$

where, $a_{1}=v+\mu ,a_{2}=rc_{s}+(1-r)c+\mu +\mu^{\prime },a_{3}=c_{r}+\mu +\mu^{\prime }.$

The characteristic polynomial for the Jacobian matrix mentioned above is:


$$Q(\lambda )=(\lambda -\mu {)}^{3}[\lambda^{4}+(2a_{1}+a_{2}+a_{3})\lambda^{3}+(a_{1}a_{3}-v\beta_{r}+a_{1}^{2}+2a_{1}a_{2}+a_{1}a_{3}+a_{2}a_{3}\;[6pt]-v\beta_{s})\lambda^{2}+(a_{1}^{2}a_{3}+a_{1}a_{2}a_{3}-a_{1}v\beta_{r}-a_{2}v\beta_{r}+a_{1}^{2}a_{2}+a_{1}a_{2}a_{3})\lambda +a_{1}^{2}a_{2}a_{3}-a_{1}a_{2}v\beta_{r}\;[6pt]-v\beta_{s}a_{1}a_{3}+v^{2}\beta_{s}\beta_{r}].$$

Then, the corresponding eigen values are:

$\lambda_{1}=\frac{-2a_{1}a_{3}}{\sqrt{a_{1}^{2}-2a_{1}a_{3}+4v\beta_{r}+a_{3}^{2}+(a_{1}+a_{3})}}(1-R_{0}^{r}),\lambda_{2}=\frac{a_{1}+a_{3}+\sqrt{a_{1}^{2}-2a_{1}a_{3}+4v\beta_{r}+a_{3}^{2}}}{-2},$

$\lambda_{3}=\frac{-2a_{1}a_{2}}{\sqrt{a_{1}^{2}-2a_{1}a_{2}+4v\beta_{s}+a_{2}^{2}+(a_{1}+a_{2})}}(1-R_{0}^{s}),\lambda_{4}=\frac{a_{1}+a_{2}+\sqrt{a_{1}^{2}-2a_{1}a_{2}+4v\beta_{s}+a_{2}^{2}}}{-2},$

$\lambda_{5}=\lambda_{6}=\lambda_{7}=-\mu$.

Obviously, if *R*_0_<1, all eigenvalues of matrix ${𝕁}_{p_{0}^{*}}$ are negative. Hence, if $\left|arg(s_{F-V})\right|>\frac{q\pi }{2}$, $P_{0}^{*}$ is locally asymptotically stable.

### 4. Parameter estimation and model fitting

In this study, we used China’s tuberculosis data from 2008 to 2023 for model fitting and parameter estimation. The information regarding TB cases can be obtained from the National Health Commission of the People’s Republic of China [37]. Parameters $c_{s},c_{r},r,$ and $\mu^{\prime }$ are referenced from the paper [35]. Parameters *μ* and *π* are obtained from the National Bureau of Statistics [38]. We obtain the estimation of parameters $\beta_{s},\beta_{r},v$ and *c* through the least squares estimate method based on the following function:


$$Q(\beta_{s},\beta_{r},v,c)=\sum_{n=1}^{16}[I_{r_{n}}-{\bar{I}}_{r_{n}}{]}^{2}.$$

Where, $I_{r_{n}}$ represents the predicted annual new infections by the model, and ${\bar{I}}_{r_{n}}$ represents the actual data of new infections each year.

**Fig 2:**
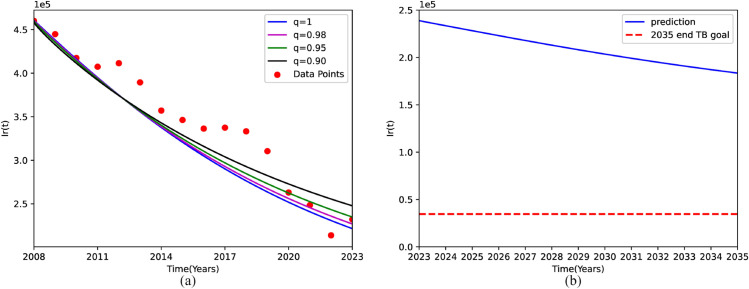
Model calibration and long-term prediction of drug-resistant tuberculosis (DR-TB) burden in China. (a) Fitting performance of the fractional-order model against observed DR-TB cases from 2008 to 2023. (b) Projected trajectory of DR-TB incidence from 2024 to 2035 under the calibrated model.

In the fractional order model, the memory index *q* is an extremely important parameter. We can adjust the value of *q* in the fractional order model to achieve the optimal fit between the data and the model. Assuming that the order *q* of the established fractional order model is set to 0.95, based on this parameter setting, Fig 2 shows that although the number of patients with DR-TB is projected to decrease gradually according to our model, it will not fall below 34,624 cases by 2035. That is to say, reducing the number of people with DR-TB to 10% of 2015 levels cannot be achieved in 2035 if no control measures are taken. All estimated parameters, as well as the fixed parameters, are listed in Table 2.

**Table 2:** Estimated model parameters.

Parameters	Biological meanings	Source
$\beta_{s}$	0.60011	Fitted
$\beta_{r}$	0.30011	Fitted
*v*	$7.5\times 10^{-5}$	Fitted
*c*	0.08	Fitted
*c*_*s*_	0.1299	[27]
*c*_*r*_	0.0493	[27]
*r*	0.85	[27]
*μ*	$6.56\times 10^{-3}$	[30]
$\mu^{\prime }$	$4.17\times 10^{-3}$	[27]
*π*	75000	[30]

## Existence and uniqueness of solutions

In this section, we transform the deterministic DR-TB model into a stochastic model by incorporating the effects of environmental white noise. Then, the associated stochastic model for DR-TB is presented as follows:

$$dS=(\pi -\mu S-\frac{\beta_{s}I_{s}}{N}S-\frac{\beta_{r}I_{r}}{N}S)dt+\sigma_{1}SdB_{1}(t),\;dE_{s}=(\frac{\beta_{s}I_{s}}{N}S-vE_{s}-\mu E_{s})dt+\sigma_{2}E_{s}dB_{2}(t),\;dI_{s}=\left\{vE_{s}-[rc_{s}+(1-r)c]I_{s}-(\mu +\mu^{{\;}^{\prime }})I_{s}\right\}dt+\sigma_{3}I_{s}dB_{3}(t),\;dR_{s}=(rc_{s}I_{s}-\mu R_{s})dt+\sigma_{4}R_{s}dB_{4}(t),\;dE_{r}=(\frac{\beta_{r}I_{r}}{N}S-vE_{r}-\mu E_{r})dt+\sigma_{5}E_{r}dB_{5}(t),\;dI_{r}=\left\{vE_{r}+(1-r)cI_{s}-(c_{r}+\mu +\mu^{{\;}^{\prime }})I_{r}\right\}d(t)+\sigma_{6}I_{r}dB_{6}(t),\;dR_{r}=(c_{r}I_{r}-\mu R_{r})dt+\sigma_{7}R_{r}dB_{7}(t),$$ (4)

where, $B_{1},B_{2},B_{3},B_{4},B_{5},B_{6},B_{7}$ are standard Brownian motion and $\sigma_{1},\sigma_{2},\sigma_{3},\sigma_{4},\sigma_{5},\sigma_{6},\sigma_{7}$ are considered to depict the corresponding intensities of the white noise. In order to prove the existence and uniqueness of the solution, the simplification of Eq (4) is given as,

$$dS=U_{1}(t,S)dt+V_{1}(t,S)dB_{1}(t),\;dE_{s}=U_{2}(t,E_{s})dt+V_{2}(t,E_{s})dB_{2}(t),\;dI_{s}=U_{3}(t,I_{s})dt+V_{3}(t,I_{s})dB_{3}(t),\;dR_{s}=U_{4}(t,R_{s})dt+V_{4}(t,R_{s})dB_{4}(t),\;dE_{r}=U_{5}(t,E_{r})dt+V_{5}(t,E_{r})dB_{5}(t),\;dI_{r}=U_{6}(t,I_{r})dt+V_{6}(t,I_{r})dB_{6}(t),\;dR_{r}=U_{7}(t,R_{r})dt+V_{7}(t,R_{r})dB_{7}(t).$$ (5)

Therefore, we convert Eq (5) into a Volterra integral operator as follows:


$$S(t)=S(0)+\int_{0}^{t}U_{1}(\xi ,S)d\xi +\int_{0}^{t}V_{1}(\xi ,S)dB_{1}(\xi ),\;E_{s}(t)=E_{s}(0)+\int_{0}^{t}U_{2}(\xi ,E_{s})d\xi +\int_{0}^{t}V_{2}(\xi ,E_{s})dB_{2}(\xi ),\;I_{s}(t)=I_{s}(0)+\int_{0}^{t}U_{3}(\xi ,I_{s})d\xi +\int_{0}^{t}V_{3}(\xi ,I_{s})dB_{3}(\xi ),\;R_{s}(t)=R_{s}(0)+\int_{0}^{t}U_{4}(\xi ,R_{s})d\xi +\int_{0}^{t}V_{4}(\xi ,R_{s})dB_{4}(\xi ),\;E_{r}(t)=E_{r}(0)+\int_{0}^{t}U_{5}(\xi ,E_{r})d\xi +\int_{0}^{t}V_{5}(\xi ,E_{r})dB_{5}(\xi ),\;I_{r}(t)=I_{r}(0)+\int_{0}^{t}U_{6}(\xi ,I_{r})d\xi +\int_{0}^{t}V_{6}(\xi ,I_{r})dB_{6}(\xi ),\;R_{r}(t)=R_{r}(0)+\int_{0}^{t}U_{7}(\xi ,R_{r})d\xi +\int_{0}^{t}V_{7}(\xi ,R_{r})dB_{7}(\xi ).$$

The following Theorem 3 proves the existence and uniqueness of solution to the DR-TB stochastic model.

**Theorem 3.**

Assume that, there exist positive constants ${𝒦}_{i}$, $\hat{{𝒦}_{i}}$ such that the following conditions [25,29] hold:

(i) ${\left|U_{i}(x,t)-U_{i}(x_{i},t)\right|}^{2}<{𝒦}_{i}{\left|x-x_{i}\right|}^{2},{\left|V_{i}(x,t)-V_{i}(x_{i},t)\right|}^{2}<\hat{{𝒦}_{i}}{\left|x-x_{i}\right|}^{2},$

(ii) $\forall (x,t)\in {\mathbb{R}}^{4}\times [0,T]$, then ${\left|U_{i}(x,t)\right|}^{2},{\left|V_{i}(x,t)\right|}^{2}<𝒦({\left|x\right|}^{2}+1),$ are satisfied for i=1,2,3,4,5,6,7.

Then, there exist a unique solution $𝒳(t)\in {\mathbb{R}}^{7}$ for Eq (4) and it belongs to $M^{2}([0,T],{\mathbb{R}}^{8})$.

**Proof.**

Using the definition of the following norm ${\left\|\psi \right\|}_{\infty }=\underset{t\in [0,T]}{sup}{\left|\psi \right|}^{2}.$ Then


$$(i){\left\|U_{1}(t,S)-U_{1}(t,S_{1})\right\|}^{2}={\left\|(\mu +\frac{\beta_{s}I_{s}}{N}+\frac{\beta_{r}I_{r}}{N})(S-S_{1})\right\|}^{2},\;\leq \underset{t\in [0,T]}{sup}{\left\|(\mu +\frac{\beta_{s}I_{s}}{N}+\frac{\beta_{r}I_{r}}{N})(S-S_{1})\right\|}^{2},\;\leq {\left\|\mu +\frac{\beta_{s}I_{s}}{N}+\frac{\beta_{r}I_{r}}{N}\right\|}_{\infty }^{2}{\left|S-S_{1}\right|}^{2},\;\leq {𝒦}_{1}{\left|S-S_{1}\right|}^{2}.\;$$

$${\left\|U_{2}(t,E_{s})-U_{2}(t,E_{s_{1}})\right\|}^{2}={\left\|(v+\mu )(E_{s}-E_{s_{1}})\right\|}^{2},\;\leq 2{\left|v+\mu \right|}^{2}{\left|E_{s}-E_{s_{1}}\right|}^{2},\;\leq {𝒦}_{2}{\left|E_{s}-E_{s_{1}}\right|}^{2}.\;{\left\|U_{3}(t,I_{s})-U_{3}(t,I_{s_{1}})\right\|}^{2}={\left\|[rc_{s}+(1-r)c+\mu +\mu^{\prime }](I_{s}-I_{s_{1}})\right\|}^{2},\;\leq 2{\left|rc_{s}+(1-r)c+\mu +\mu^{\prime }\right|}^{2}{\left|I_{s}-I_{s_{1}}\right|}^{2},\;\leq {𝒦}_{3}{\left|I_{s}-I_{s_{1}}\right|}^{2}.\;$$

$${\left\|U_{4}(t,R_{s})-U_{4}(t,R_{s_{1}})\right\|}^{2}={\left\|\mu (R_{s}-R_{s_{1}})\right\|}^{2},\;\leq 2{\left|\mu \right|}^{2}{\left|R_{s}-R_{s_{1}}\right|}^{2},\;\leq {𝒦}_{4}{\left|R_{s}-R_{s_{1}}\right|}^{2}.\;{\left\|U_{5}(t,E_{r})-U_{5}(t,E_{r_{1}})\right\|}^{2}={\left\|(v+\mu )(E_{r}-E_{r_{1}})\right\|}^{2},\;\leq 2{\left|(v+\mu )\right|}^{2}{\left|E_{r}-E_{r_{1}}\right|}^{2},\;\leq {𝒦}_{5}{\left|E_{r}-E_{r_{1}}\right|}^{2}.\;{\left\|U_{6}(t,I_{r})-U_{6}(t,I_{r_{1}})\right\|}^{2}={\left\|(c_{r}+\mu +\mu^{\prime })(I_{r}-I_{r_{1}})\right\|}^{2},\;\leq 2{\left|(c_{r}+\mu +\mu^{\prime })\right|}^{2}{\left|I_{r}-I_{r_{1}}\right|}^{2},\;\leq {𝒦}_{6}{\left|I_{r}-I_{r_{1}}\right|}^{2}.\;{\left\|U_{7}(t,R_{r})-U_{7}(t,R_{r_{1}})\right\|}^{2}={\left\|\mu (R_{r}-R_{r_{1}})\right\|}^{2},\;\leq 2{\left|\mu \right|}^{2}{\left|R_{r}-R_{r_{1}}\right|}^{2},\;\leq {𝒦}_{7}{\left|R_{r}-R_{r_{1}}\right|}^{2}.$$

where, ${𝒦}_{1}={\left\|\mu +\frac{\beta_{s}I_{s}}{N}+\frac{\beta_{r}I_{r}}{N}\right\|}_{\infty }^{2},{𝒦}_{2}=2{\left|v+\mu \right|}^{2},{𝒦}_{3}=2{\left|rc_{s}+(1-r)c+\mu +\mu^{\prime }\right|}^{2},{𝒦}_{4}=2{\left|\mu \right|}^{2},{𝒦}_{5}=2{\left|v+\mu \right|}^{2},{𝒦}_{6}=2{\left|c_{r}+\mu +\mu^{\prime }\right|}^{2},{𝒦}_{7}=2{\left|\mu \right|}^{2}.$


$${\left\|V_{1}(t,S)-V_{1}(t,S_{1})\right\|}^{2}={\left|\sigma_{1}(S-S_{1})\right|}^{2}\leq \frac{3}{2}\sigma_{1}^{2}{\left|S-S_{1}\right|}^{2}\leq {\hat{𝒦}}_{1}{\left|S-S_{1}\right|}^{2},\;{\left\|V_{2}(t,E_{s})-V_{2}(t,E_{s_{1}})\right\|}^{2}={\left|\sigma_{2}(E_{s}-E_{s_{1}})\right|}^{2}\leq \frac{3}{2}\sigma_{2}^{2}{\left|E_{s}-E_{s_{1}}\right|}^{2}\leq {\hat{𝒦}}_{2}{\left|E_{s}-E_{s_{1}}\right|}^{2},\;{\left\|V_{3}(t,I_{s})-V_{3}(t,I_{s_{1}})\right\|}^{2}={\left|\sigma_{3}(I_{s}-I_{s_{1}})\right|}^{2}\leq \frac{3}{2}\sigma_{3}^{2}{\left|I_{s}-I_{s_{1}}\right|}^{2}\leq {\hat{𝒦}}_{3}{\left|I_{s}-I_{s_{1}}\right|}^{2},\;{\left\|V_{4}(t,R_{s})-V_{4}(t,R_{s_{1}})\right\|}^{2}={\left|\sigma_{4}(R_{s}-R_{s_{1}})\right|}^{2}\leq \frac{3}{2}\sigma_{4}^{2}{\left|R_{s}-R_{s_{1}}\right|}^{2}\leq {\hat{𝒦}}_{4}{\left|R_{s}-R_{s_{1}}\right|}^{2},\;{\left\|V_{5}(t,E_{r})-V_{5}(t,E_{r_{1}})\right\|}^{2}={\left|\sigma_{5}(E_{r}-E_{r_{1}})\right|}^{2}\leq \frac{3}{2}\sigma_{5}^{2}{\left|E_{r}-E_{r_{1}}\right|}^{2}\leq {\hat{𝒦}}_{5}{\left|E_{r}-E_{r_{1}}\right|}^{2},\;{\left\|V_{6}(t,I_{r})-V_{6}(t,I_{r_{1}})\right\|}^{2}={\left|\sigma_{6}(I_{r}-I_{r_{1}})\right|}^{2}\leq \frac{3}{2}\sigma_{6}^{2}{\left|I_{r}-I_{r_{1}}\right|}^{2}\leq {\hat{𝒦}}_{6}{\left|I_{r}-I_{r_{1}}\right|}^{2},\;{\left\|V_{7}(t,R_{r})-V_{7}(t,R_{r_{1}})\right\|}^{2}={\left|\sigma_{7}(R_{r}-R_{r_{1}})\right|}^{2}\leq \frac{3}{2}\sigma_{7}^{2}{\left|R_{r}-R_{r_{1}}\right|}^{2}\leq {\hat{𝒦}}_{7}{\left|R_{r}-R_{r_{1}}\right|}^{2},$$

where, ${\hat{𝒦}}_{1}=\frac{3}{2}\sigma_{1}^{2},{\hat{𝒦}}_{2}=\frac{3}{2}\sigma_{2}^{2},{\hat{𝒦}}_{3}=\frac{3}{2}\sigma_{3}^{2},{\hat{𝒦}}_{4}=\frac{3}{2}\sigma_{4}^{2},{\hat{𝒦}}_{5}=\frac{3}{2}\sigma_{5}^{2},{\hat{𝒦}}_{6}=\frac{3}{2}\sigma_{6}^{2},{\hat{𝒦}}_{7}=\frac{3}{2}\sigma_{7}^{2}$.

(ii)


$${\left|U_{1}(t,S)\right|}^{2}={\left|\pi -\mu S-\frac{\beta_{s}I_{s}}{N}S-\frac{\beta_{r}I_{r}}{N}S\right|}^{2},\;\leq 3[\pi^{2}+(\frac{\beta_{s}\left|I_{s}\right|}{N}+\frac{\beta_{r}\left|I_{r}\right|}{N}{)}^{2}{\left|S\right|}^{2}+\mu^{2}{\left|S\right|}^{2}],\;\leq 3[\pi^{2}+2(\frac{\beta_{s}^{2}{\left|I_{s}\right|}^{2}}{N^{2}}+\frac{\beta_{r}^{2}{\left|I_{r}\right|}^{2}}{N^{2}}){\left|S\right|}^{2}+\mu^{2}{\left|S\right|}^{2}],\;\leq 3[\pi^{2}+2(\frac{\beta_{s}^{2}}{N^{2}}\underset{0\leq t\leq T}{sup}{\left|I_{s}\right|}^{2}+\frac{\beta_{r}^{2}}{N^{2}}\underset{0\leq t\leq T}{sup}{\left|I_{r}\right|}^{2}){\left|S\right|}^{2}+\mu^{2}{\left|S\right|}^{2}],\;\leq 3[\pi^{2}+2(\frac{\beta_{s}^{2}}{N^{2}}{\left\|I_{s}^{2}\right\|}_{\infty }+\frac{\beta_{r}^{2}}{N^{2}}{\left\|I_{r}^{2}\right\|}_{\infty }+\mu^{2}){\left|S\right|}^{2}],\;\leq 3\pi^{2}[1+\frac{2(\frac{\beta_{s}^{2}}{N^{2}}{\left\|I_{s}^{2}\right\|}_{\infty }+\frac{\beta_{r}^{2}}{N^{2}}{\left\|I_{r}^{2}\right\|}_{\infty }+\mu^{2})}{\pi^{2}}{\left|S\right|}^{2}].$$

${\left|U_{1}(t,S)\right|}^{2}\leq {𝒦}^{1}({\left|S\right|}^{2}+1)$, under the condition $\frac{2(\frac{\beta_{s}^{2}}{N^{2}}{\left\|I_{s}^{2}\right\|}_{\infty }+\frac{\beta_{r}^{2}}{N^{2}}{\left\|I_{r}^{2}\right\|}_{\infty }+\mu^{2})}{\pi^{2}}<1$ .


$${\left|U_{2}(t,E_{s})\right|}^{2}={\left|\frac{\beta_{s}I_{s}}{N}S-vE_{s}-\mu E_{s}\right|}^{2},\;\leq 2[\frac{\beta_{s}^{2}{\left|I_{s}\right|}^{2}}{N^{2}}+2(v^{2}+\mu^{2}){\left|E_{s}\right|}^{2}],\;\leq 2[\frac{\beta_{s}^{2}}{N^{2}}\underset{0\leq t\leq T}{sup}{\left|I_{s}\right|}^{2}+2(v^{2}+\mu^{2}){\left|E_{s}\right|}^{2}],\;\leq 2[\frac{\beta_{s}^{2}}{N^{2}}{\left\|I_{s}^{2}\right\|}_{\infty }+2(v^{2}+\mu^{2}){\left|E_{s}\right|}^{2}],\;\leq 2\frac{\beta_{s}^{2}{\left\|I_{s}^{2}\right\|}_{\infty }}{N^{2}}[1+\frac{2(v^{2}+\mu^{2})N^{2}}{\beta_{s}^{2}{\left\|I_{s}^{2}\right\|}_{\infty }}{\left|E_{s}\right|}^{2}].$$

${\left|U_{2}(t,E_{s})\right|}^{2}\leq {𝒦}^{2}({\left|E_{s}\right|}^{2}+1),$ under the condition $\frac{2(v^{2}+\mu^{2})N^{2}}{\beta_{s}^{2}{\left\|I_{s}^{2}\right\|}_{\infty }}<1$.


$${\left|U_{3}(t,I_{s})\right|}^{2}={\left|vE_{s}-[rc_{s}+(1-r)c]I_{s}-(\mu +\mu^{\prime })I_{s}\right|}^{2},\;\leq 3\left(v^{2}{\left|E_{s}\right|}^{2}+[rc_{s}+(1-r)c{]}^{2}{\left|I_{s}\right|}^{2}+(\mu +\mu^{{\;}^{\prime }}){\left|I_{s}\right|}^{2}\right),\;\leq 3\left(v^{2}{\left|E_{s}\right|}^{2}+2[r^{2}c_{s}^{2}+(1-r{)}^{2}c^{2}]{\left|I_{s}\right|}^{2}+2(\mu^{2}+\mu^{{\;}^{\prime }2}){\left|I_{s}\right|}^{2}\right),\;\leq 3\left(v^{2}\underset{0\leq t\leq T}{sup}{\left|E_{s}\right|}^{2}+2[r^{2}c_{s}^{2}+(1-r{)}^{2}c^{2}+\mu^{2}+\mu^{{\;}^{\prime }2}]{\left|I_{s}\right|}^{2}\right),\;\leq 3\left(v^{2}{\left\|E_{s}^{2}\right\|}_{\infty }+2[r^{2}c_{s}^{2}+(1-r{)}^{2}c^{2}+\mu^{2}+\mu^{{\;}^{\prime }2}]{\left|I_{s}\right|}^{2}\right),\;\leq 3v^{2}{\left\|E_{s}^{2}\right\|}_{\infty }\left(1+\frac{2[r^{2}c_{s}^{2}+(1-r{)}^{2}c^{2}+\mu^{2}+\mu^{{\;}^{\prime }2}]}{v^{2}{\left\|E_{s}^{2}\right\|}_{\infty }}{\left|I_{s}\right|}^{2}\right).$$

${\left|U_{3}(t,I_{s})\right|}^{2}\leq {𝒦}^{3}({\left|I_{s}\right|}^{2}+1)$, under the condition $\frac{2[r^{2}c_{s}^{2}+(1-r{)}^{2}c^{2}+\mu^{2}+\mu^{{\;}^{\prime }2}]}{v^{2}{\left\|E_{s}^{2}\right\|}_{\infty }}<1$.


$${\left|U_{4}(t,R_{s})\right|}^{2}={\left|rc_{s}I_{s}-\mu R_{s}\right|}^{2},\;\leq 2(r^{2}c_{s}^{2}{\left|I_{s}\right|}^{2}+\mu^{2}{\left|R_{s}\right|}^{2}),\;\leq 2\left(r^{2}c_{s}^{2}\underset{0\leq t\leq T}{sup}{\left|I_{s}\right|}^{2}+\mu^{2}{\left|R_{s}\right|}^{2}\right),\;\leq 2(r^{2}c_{s}^{2}{\left\|I_{s}^{2}\right\|}_{\infty }+\mu^{2}{\left|R_{s}\right|}^{2}),\;\leq 2r^{2}c_{s}^{2}{\left\|I_{s}^{2}\right\|}_{\infty }(1+\frac{\mu^{2}}{r^{2}c_{s}^{2}{\left\|I_{s}^{2}\right\|}_{\infty }}{\left|R_{s}\right|}^{2}).$$

${\left|U_{4}(t,R_{s})\right|}^{2}\leq {𝒦}^{4}({\left|R_{s}\right|}^{2}+1)$, under the condition $\frac{\mu^{2}}{r^{2}c_{s}^{2}{\left\|I_{s}^{2}\right\|}_{\infty }}<1$.


$${\left|U_{5}(t,E_{r})\right|}^{2}={\left|\frac{\beta_{r}I_{r}}{N}S-vE_{r}-\mu E_{r}\right|}^{2},\;\leq 3(\frac{\beta_{r}^{2}{\left|I_{r}\right|}^{2}{\left|S\right|}^{2}}{N^{2}}+v^{2}{\left|E_{r}\right|}^{2}+\mu^{2}{\left|E_{r}\right|}^{2}),\;\leq 3\left(\frac{\beta_{r}^{2}}{N^{2}}\underset{0\leq t\leq T}{sup}{\left|I_{r}\right|}^{2}\underset{0\leq t\leq T}{sup}{\left|S\right|}^{2}+(v^{2}+\mu^{2}){\left|E_{r}\right|}^{2}\right),\;\leq 3\left(\frac{\beta_{r}^{2}}{N^{2}}{\left\|I_{r}\right\|}_{\infty }^{2}{\left\|S\right\|}_{\infty }^{2}+(v^{2}+\mu^{2}){\left|E_{r}\right|}^{2}\right),\;\leq 3\frac{\beta_{r}^{2}{\left\|I_{r}\right\|}_{\infty }^{2}{\left\|S\right\|}_{\infty }^{2}}{N^{2}}\left(1+\frac{(v^{2}+\mu^{2})N^{2}}{\beta_{r}^{2}{\left\|I_{r}\right\|}_{\infty }^{2}{\left\|S\right\|}_{\infty }^{2}}{\left|E_{r}\right|}^{2}\right).$$

${\left|U_{5}(t,E_{r})\right|}^{2}\leq {𝒦}^{5}({\left|E_{r}\right|}^{2}+1),$ under the condition $\frac{(v^{2}+\mu^{2})N^{2}}{\beta_{r}^{2}{\left\|I_{r}\right\|}_{\infty }^{2}{\left\|S\right\|}_{\infty }^{2}}<1$.


$${\left|U_{6}(t,I_{r})\right|}^{2}={\left|vE_{r}+(1-r)cI_{s}-(c_{r}+\mu +\mu^{\prime })I_{r}\right|}^{2},\;\leq 3[v^{2}{\left|E_{r}\right|}^{2}+(1-r{)}^{2}c^{2}{\left|I_{s}\right|}^{2}+(c_{r}+\mu +\mu^{{\;}^{\prime }}{)}^{2}{\left|I_{r}\right|}^{2}],\;\leq 3[v^{2}{\left|E_{r}\right|}^{2}+(1-r{)}^{2}c^{2}{\left|I_{s}\right|}^{2}+3(c_{r}^{2}+\mu^{2}+\mu^{{\;}^{\prime }2}){\left|I_{r}\right|}^{2}],\;\leq 3\left(v^{2}\underset{0\leq t\leq T}{sup}{\left|E_{r}\right|}^{2}+(1-r{)}^{2}c^{2}\underset{0\leq t\leq T}{sup}{\left|I_{s}\right|}^{2}+3(c_{r}^{2}+\mu^{2}+\mu^{{\;}^{\prime }2}){\left|I_{r}\right|}^{2}\right),\;\leq 3\left(v^{2}{\left\|E_{r}\right\|}_{\infty }^{2}+(1-r{)}^{2}c^{2}{\left\|I_{s}\right\|}_{\infty }^{2}+3(c_{r}^{2}+\mu^{2}+\mu^{{\;}^{\prime }2}){\left|I_{r}\right|}^{2}\right),\;\leq 3(v^{2}{\left\|E_{r}\right\|}_{\infty }^{2}+(1-r{)}^{2}c^{2}{\left\|I_{s}^{2}\right\|}_{\infty })\left(1+\frac{3(c_{r}^{2}+\mu^{2}+\mu^{{\;}^{\prime }2})}{v^{2}{\left\|E_{r}\right\|}_{\infty }^{2}+(1-r{)}^{2}c^{2}{\left\|I_{s}^{2}\right\|}_{\infty }}{\left|I_{r}\right|}^{2}\right).$$

${\left|U_{6}(t,I_{r})\right|}^{2}\leq {𝒦}^{6}({\left|I_{r}\right|}^{2}+1),$ under the condition $\frac{3(c_{r}^{2}+\mu^{2}+\mu^{{\;}^{\prime }2})}{v^{2}{\left\|E_{r}\right\|}_{\infty }^{2}+(1-r{)}^{2}c^{2}{\left\|I_{s}^{2}\right\|}_{\infty }}<1$.


$${\left|U_{7}(t,R_{r})\right|}^{2}={\left|c_{r}I_{r}-\mu R_{r}\right|}^{2},\;\leq 2(c_{r}^{2}{\left|I_{r}\right|}^{2}+\mu^{2}{\left|R_{r}\right|}^{2}),\;\leq 2\left(c_{r}^{2}\underset{0\leq t\leq T}{sup}{\left|I_{r}\right|}^{2}+\mu^{2}{\left|R_{r}\right|}^{2}\right),\;\leq 2\left(c_{r}^{2}{\left\|I_{r}^{2}\right\|}_{\infty }+\mu^{2}{\left|R_{r}\right|}^{2}\right),\;\leq 2c_{r}^{2}{\left\|I_{r}^{2}\right\|}_{\infty }\left(1+\frac{\mu^{2}}{c_{r}^{2}{\left\|I_{r}^{2}\right\|}_{\infty }}{\left|R_{r}\right|}^{2}\right).$$

${\left|U_{7}(t,R_{r})\right|}^{2}\leq {𝒦}^{7}({\left|R_{r}\right|}^{2}+1)$, under the condition $\frac{\mu^{2}}{c_{r}^{2}{\left\|I_{r}^{2}\right\|}_{\infty }}<1$.


$${\left|V_{1}(t,S)\right|}^{2}={\left|\sigma_{1}S\right|}^{2}\leq \sigma_{1}^{2}{\left|S\right|}^{2}\leq \sigma_{1}^{2}({\left|S\right|}^{2}+1)\leq \hat{{𝒦}^{1}}({\left|S\right|}^{2}+1),\;{\left|V_{2}(t,E_{s})\right|}^{2}={\left|\sigma_{2}E_{s}\right|}^{2}\leq \sigma_{2}^{2}{\left|E_{s}\right|}^{2}\leq \sigma_{2}^{2}({\left|E_{s}\right|}^{2}+1)\leq \hat{{𝒦}^{2}}({\left|E_{s}\right|}^{2}+1),\;{\left|V_{3}(t,I_{s})\right|}^{2}={\left|\sigma_{3}I_{s}\right|}^{2}\leq \sigma_{3}^{2}{\left|I_{s}\right|}^{2}\leq \sigma_{3}^{2}({\left|I_{s}\right|}^{2}+1)\leq \hat{{𝒦}^{3}}({\left|I_{s}\right|}^{2}+1),\;{\left|V_{4}(t,R_{s})\right|}^{2}={\left|\sigma_{4}R_{s}\right|}^{2}\leq \sigma_{4}^{2}{\left|R_{s}\right|}^{2}\leq \sigma_{4}^{2}({\left|R_{s}\right|}^{2}+1)\leq \hat{{𝒦}^{4}}({\left|R_{s}\right|}^{2}+1),\;{\left|V_{5}(t,E_{r})\right|}^{2}={\left|\sigma_{5}E_{r}\right|}^{2}\leq \sigma_{5}^{2}{\left|E_{r}\right|}^{2}\leq \sigma_{5}^{2}({\left|E_{r}\right|}^{2}+1)\leq \hat{{𝒦}^{5}}({\left|E_{r}\right|}^{2}+1),\;{\left|V_{6}(t,I_{r})\right|}^{2}={\left|\sigma_{6}I_{r}\right|}^{2}\leq \sigma_{6}^{2}{\left|I_{r}\right|}^{2}\leq \sigma_{6}^{2}({\left|I_{r}\right|}^{2}+1)\leq \hat{{𝒦}^{6}}({\left|I_{r}\right|}^{2}+1),\;{\left|V_{7}(t,R_{r})\right|}^{2}={\left|\sigma_{7}R_{r}\right|}^{2}\leq \sigma_{7}^{2}{\left|R_{r}\right|}^{2}\leq \sigma_{7}^{2}({\left|R_{r}\right|}^{2}+1)\leq \hat{{𝒦}^{7}}({\left|R_{r}\right|}^{2}+1),$$

where, $\hat{{𝒦}^{1}}=\sigma_{1}^{2},\hat{{𝒦}^{2}}=\sigma_{2}^{2},\hat{{𝒦}^{3}}=\sigma_{3}^{2},\hat{{𝒦}^{4}}=\sigma_{4}^{2},\hat{{𝒦}^{5}}=\sigma_{5}^{2},\hat{{𝒦}^{6}}=\sigma_{6}^{2},\hat{{𝒦}^{7}}=\sigma_{7}^{2}$.

Hence, if their growth condition exists such that


$$\max\left\{\begin{array}{c} \frac{2\left(\frac{\beta_{s}^{2}}{N^{2}}{\left\|I_{s}^{2}\right\|}_{\infty }+\frac{\beta_{r}^{2}}{N^{2}}{\left\|I_{r}^{2}\right\|}_{\infty }+\mu^{2}\right)}{\pi^{2}},\frac{2(v^{2}+\mu^{2})N^{2}}{\beta_{s}^{2}{\left\|I_{s}^{2}\right\|}_{\infty }},\frac{2\left[r^{2}c_{s}^{2}+(1-r{)}^{2}c^{2}+\mu^{2}+\mu^{\prime 2}\right]}{v^{2}{\left\|E_{s}^{2}\right\|}_{\infty }},\\ \frac{\mu^{2}}{r^{2}c_{s}^{2}{\left\|I_{s}^{2}\right\|}_{\infty }},\frac{(v^{2}+\mu^{2})N^{2}}{\beta_{r}^{2}{\left\|I_{r}^{2}\right\|}_{\infty }{\left\|S\right\|}_{\infty }^{2}},\frac{3(c_{r}^{2}+\mu^{2}+\mu^{\prime 2})}{v^{2}{\left\|E_{r}^{2}\right\|}_{\infty }+(1-r{)}^{2}c^{2}{\left\|I_{s}^{2}\right\|}_{\infty }},\frac{\mu^{2}}{c_{r}^{2}{\left\|I_{r}^{2}\right\|}_{\infty }} \end{array}\right\}<1.$$

Then the model system has a unique solution.

## Numerical simulation for the stochastic model

In this section, we introduce a stochastic model that incorporates the Atangana-Baleanu operator. The model is described as:

$${\;}_{0}^{ABC}D_{t}^{q}S(t)=(\pi -\mu S-\frac{\beta_{s}I_{s}}{N}S-\frac{\beta_{r}I_{r}}{N}S)+\sigma_{1}SB_{1}^{\prime }(t),\;{\;}_{0}^{ABC}D_{t}^{q}E_{s}(t)=(\frac{\beta_{s}I_{s}}{N}S-vE_{s}-\mu E_{s})+\sigma_{2}E_{s}B_{2}^{\prime }(t),\;{\;}_{0}^{ABC}D_{t}^{q}I_{s}(t)=\left\{vE_{s}-[rc_{s}+(1-r)c]I_{s}-(\mu +\mu^{{\;}^{\prime }})I_{s}\right\}+\sigma_{3}I_{s}B_{3}^{\prime }(t),\;{\;}_{0}^{ABC}D_{t}^{q}R_{s}(t)=(rc_{s}I_{s}-\mu R_{s})+\sigma_{4}R_{s}B_{4}^{\prime }(t),\;{\;}_{0}^{ABC}D_{t}^{q}E_{r}(t)=(\frac{\beta_{r}I_{r}}{N}S-vE_{r}-\mu E_{r})+\sigma_{5}E_{r}B_{5}^{\prime }(t),\;{\;}_{0}^{ABC}D_{t}^{q}I_{r}(t)=\left\{vE_{r}+(1-r)cI_{s}-(c_{r}+\mu +\mu^{{\;}^{\prime }})I_{r}\right\}+\sigma_{6}I_{r}B_{6}^{\prime }(t),\;{\;}_{0}^{ABC}D_{t}^{q}R_{r}(t)=(c_{r}I_{r}-\mu R_{r})+\sigma_{7}R_{r}B_{7}^{\prime }(t)$$ (6)

For simplification purpose, the Eq (6) is organized as:

$${\;}_{0}^{ABC}D_{t}^{q}S(t)=S(t,S,E_{s},I_{s},R_{s},E_{r},I_{r},R_{r})+\sigma_{1}{ℋ}_{1}(t)B_{1}^{\prime }(t),\;{\;}_{0}^{ABC}D_{t}^{q}E_{s}(t)=E_{s}(t,S,E_{s},I_{s},R_{s},E_{r},I_{r},R_{r})+\sigma_{2}{ℋ}_{2}(t)B_{2}^{\prime }(t),\;{\;}_{0}^{ABC}D_{t}^{q}I_{s}(t)=I_{s}(t,S,E_{s},I_{s},R_{s},E_{r},I_{r},R_{r})+\sigma_{3}{ℋ}_{3}(t)B_{3}^{\prime }(t),\;{\;}_{0}^{ABC}D_{t}^{q}R_{s}(t)=R_{s}(t,S,E_{s},I_{s},R_{s},E_{r},I_{r},R_{r})+\sigma_{4}{ℋ}_{4}(t)B_{4}^{\prime }(t),\;{\;}_{0}^{ABC}D_{t}^{q}E_{r}(t)=E_{r}(t,S,E_{s},I_{s},R_{s},E_{r},I_{r},R_{r})+\sigma_{5}{ℋ}_{5}(t)B_{5}^{\prime }(t),\;{\;}_{0}^{ABC}D_{t}^{q}I_{r}(t)=I_{r}(t,S,E_{s},I_{s},R_{s},E_{r},I_{r},R_{r})+\sigma_{6}{ℋ}_{6}(t)B_{6}^{\prime }(t),\;{\;}_{0}^{ABC}D_{t}^{q}R_{r}(t)=R_{r}(t,S,E_{s},I_{s},R_{s},E_{r},I_{r},R_{r})+\sigma_{7}{ℋ}_{7}(t)B_{7}^{\prime }(t).$$ (7)

Here, we illustrate the numerical scheme of the Atangana-Baleanu derivative operator in Eq (7) by the Newton polynomial interpolation technique [39]. The numerical scheme for the corresponding state variables is given as.


$$S(t_{n+1})=S(0)+\frac{1-q}{ABC(q)}S(t_{n},S^{n},E_{s}^{n},I_{s}^{n},R_{s}^{n},E_{r}^{n},I_{r}^{n},R_{r}^{n})\;+\frac{q(△t{)}^{q}}{ABC(q)\Gamma (q+1)}\sum_{m=2}^{n}S(t_{m-2},S^{m-2},E_{s}^{m-2},I_{s}^{m-2},R_{s}^{m-2},E_{r}^{m-2},I_{r}^{m-2},R_{r}^{m-2})\;\times \Pi +\frac{q(△t{)}^{q}}{ABC(q)\Gamma (q+2)}\sum_{m=2}^{n}[S(t_{m-1},S^{m-1},E_{s}^{m-1},I_{s}^{m-1},R_{s}^{m-1},E_{r}^{m-1},I_{r}^{m-1},\;R_{r}^{m-1})-S(t_{m-2},S^{m-2},E_{s}^{m-2},I_{s}^{m-2},R_{s}^{m-2},E_{r}^{m-2},I_{r}^{m-2},R_{r}^{m-2})]\times \Theta \;+\frac{q(△t{)}^{q}}{2ABC(q)\Gamma (q+3)}\sum_{m=2}^{n}[S(t_{m},S^{m},E_{s}^{m},I_{s}^{m},R_{s}^{m},E_{r}^{m},I_{r}^{m},R_{r}^{m})-2S(t_{m-1},S^{m-1},\;E_{s}^{m-1},I_{s}^{m-1},R_{s}^{m-1},E_{r}^{m-1},I_{r}^{m-1},R_{r}^{m-1})+S(t_{m-2},S^{m-2},E_{s}^{m-2},I_{s}^{m-2},R_{s}^{m-2},\;E_{r}^{m-2},I_{r}^{m-2},R_{r}^{m-2})]\times \Omega +\frac{q(△t{)}^{q}}{ABC(q)\Gamma (q+1)}\sum_{m=2}^{n}\sigma_{1}{ℋ}_{1}(t_{m-2},S^{m-2})(B_{1}(t_{m-1})\;-B_{1}(t_{m-2}))\times \Pi +\frac{q(△t{)}^{q}}{ABC(q)\Gamma (q+2)}\sum_{m=2}^{n}[\sigma_{1}{ℋ}_{1}(t_{m-1},S^{m-1})(B_{1}(t_{m})-B_{1}(t_{m-1}))\;-\sigma_{1}{ℋ}_{1}(t_{m-2},S^{m-2})(B_{1}(t_{m-1})-B_{1}(t_{m-2}))]\times \Theta +\frac{q(△t{)}^{q}}{2ABC(q)\Gamma (q+3)}\sum_{m=2}^{n}[\sigma_{1}\;{ℋ}_{1}(t_{m},S^{m})(B_{1}(t_{m+1})-B_{1}(t_{m}))-2\sigma_{1}{ℋ}_{1}(t_{m-1},S^{m-1})(B_{1}(t_{m})-B_{1}(t_{m-1}))\;+\sigma_{1}{ℋ}_{1}(t_{m-2},S^{m-2})(B_{1}(t_{m-1})-B_{1}(t_{m-2}))]\times \Omega .$$

$$E_{s}(t_{n+1})=E_{s}(0)+\frac{1-q}{ABC(q)}E_{s}(t_{n},S^{n},E_{s}^{n},I_{s}^{n},R_{s}^{n},E_{r}^{n},I_{r}^{n},R_{r}^{n})\;+\frac{q(△t{)}^{q}}{ABC(q)\Gamma (q+1)}\sum_{m=2}^{n}E_{s}(t_{m-2},S^{m-2},E_{s}^{m-2},I_{s}^{m-2},R_{s}^{m-2},E_{r}^{m-2},I_{r}^{m-2},R_{r}^{m-2})\;\times \Pi +\frac{q(△t{)}^{q}}{ABC(q)\Gamma (q+2)}\sum_{m=2}^{n}[E_{s}(t_{m-1},S^{m-1},E_{s}^{m-1},I_{s}^{m-1},R_{s}^{m-1},E_{r}^{m-1},I_{r}^{m-1},\;R_{r}^{m-1}-E_{s}(t_{m-2},S^{m-2},E_{s}^{m-2},I_{s}^{m-2},R_{s}^{m-2},E_{r}^{m-2},I_{r}^{m-2},R_{r}^{m-2})]\times \Theta \;+\frac{q(△t{)}^{q}}{2ABC(q)\Gamma (q+3)}\sum_{m=2}^{n}[E_{s}(t_{m},S^{m},E_{s}^{m},I_{s}^{m},R_{s}^{m},E_{r}^{m},I_{r}^{m},R_{r}^{m})-2E_{s}(t_{m-1},\;S^{m-1},E_{s}^{m-1},I_{s}^{m-1},R_{s}^{m-1},E_{r}^{m-1},I_{r}^{m-1},R_{r}^{m-1})+E_{s}(t_{m-2},S^{m-2},E_{s}^{m-2},I_{s}^{m-2},\;R_{s}^{m-2},E_{r}^{m-2},I_{r}^{m-2},R_{r}^{m-2})]\times \Omega +\frac{q(△t{)}^{q}}{ABC(q)\Gamma (q+1)}\sum_{m=2}^{n}\sigma_{2}{ℋ}_{2}(t_{m-2},E_{s}^{m-2})\;(B_{2}(t_{m-1})-B_{2}(t_{m-2}))\times \Pi +\frac{q(△t{)}^{q}}{ABC(q)\Gamma (q+2)}\sum_{m=2}^{n}[\sigma_{2}{ℋ}_{2}(t_{m-1},E_{s}^{m-1})(B_{2}(t_{m})\;-B_{2}(t_{m-1}))-\sigma_{2}{ℋ}_{2}(t_{m-2},E_{s}^{m-2})(B_{2}(t_{m-1})-B_{2}(t_{m-2}))]\times \Theta \;+\frac{q(△t{)}^{q}}{2ABC(q)\Gamma (q+3)}\sum_{m=2}^{n}[\sigma_{2}{ℋ}_{2}(t_{m},E_{s}^{m})(B_{2}(t_{m+1})-B_{2}(t_{m}))-2\sigma_{2}{ℋ}_{2}(t_{m-1},\;E_{s}^{m-1})(B_{2}(t_{m})-B_{2}(t_{m-1}))+\sigma_{2}{ℋ}_{2}(t_{m-2},E_{s}^{m-2})(B_{2}(t_{m-1})-B_{2}(t_{m-2}))]\times \Omega .$$

$$I_{s}(t_{n+1})=I_{s}(0)+\frac{1-q}{ABC(q)}I_{s}(t_{n},S^{n},E_{s}^{n},I_{s}^{n},R_{s}^{n},E_{r}^{n},I_{r}^{n},R_{r}^{n})\;+\frac{q(△t{)}^{q}}{ABC(q)\Gamma (q+1)}\sum_{m=2}^{n}I_{s}(t_{m-2},S^{m-2},E_{s}^{m-2},I_{s}^{m-2},R_{s}^{m-2},E_{r}^{m-2},I_{r}^{m-2},R_{r}^{m-2})\;\times \Pi +\frac{q(△t{)}^{q}}{ABC(q)\Gamma (q+2)}\sum_{m=2}^{n}[I_{s}(t_{m-1},S^{m-1},E_{s}^{m-1},I_{s}^{m-1},R_{s}^{m-1},E_{r}^{m-1},I_{r}^{m-1},\;R_{r}^{m-1})-I_{s}(t_{m-2},S^{m-2},E_{s}^{m-2},I_{s}^{m-2},R_{s}^{m-2},E_{r}^{m-2},I_{r}^{m-2},R_{r}^{m-2})]\times \Theta \;+\frac{q(△t{)}^{q}}{2ABC(q)\Gamma (q+3)}\sum_{m=2}^{n}[I_{s}(t_{m},S^{m},E_{s}^{m},I_{s}^{m},R_{s}^{m},E_{r}^{m},I_{r}^{m},R_{r}^{m})-2I_{s}(t_{m-1},\;S^{m-1},E_{s}^{m-1},I_{s}^{m-1},R_{s}^{m-1},E_{r}^{m-1},I_{r}^{m-1},R_{r}^{m-1})+E_{s}(t_{m-2},S^{m-2},E_{s}^{m-2},I_{s}^{m-2},\;R_{s}^{m-2},E_{r}^{m-2},I_{r}^{m-2},R_{r}^{m-2})]\times \Omega +\frac{q(△t{)}^{q}}{ABC(q)\Gamma (q+1)}\sum_{m=2}^{n}\sigma_{3}{ℋ}_{3}(t_{m-2},I_{s}^{m-2})\;(B_{3}(t_{m-1})-B_{3}(t_{m-2}))\times \Pi +\frac{q(△t{)}^{q}}{ABC(q)\Gamma (q+2)}\sum_{m=2}^{n}[\sigma_{3}{ℋ}_{3}(t_{m-1},I_{s}^{m-1})(B_{3}(t_{m})\;-B_{3}(t_{m-1}))-\sigma_{3}{ℋ}_{3}(t_{m-2},I_{s}^{m-2})(B_{3}(t_{m-1})-B_{3}(t_{m-2}))]\times \Theta \;+\frac{q(△t{)}^{q}}{2ABC(q)\Gamma (q+3)}\sum_{m=2}^{n}[\sigma_{3}{ℋ}_{3}(t_{m},I_{s}^{m})(B_{3}(t_{m+1})-B_{3}(t_{m}))-2\sigma_{3}{ℋ}_{3}(t_{m-1},\;I_{s}^{m-1})(B_{3}(t_{m})-B_{3}(t_{m-1}))+\sigma_{3}{ℋ}_{3}(t_{m-2},I_{s}^{m-2})(B_{3}(t_{m-1})-B_{3}(t_{m-2}))]\times \Omega .\;R_{s}(t_{n+1})=R_{s}(0)+\frac{1-q}{ABC(q)}R_{s}(t_{n},S^{n},E_{s}^{n},I_{s}^{n},R_{s}^{n},E_{r}^{n},I_{r}^{n},R_{r}^{n})\;+\frac{q(△t{)}^{q}}{ABC(q)\Gamma (q+1)}\sum_{m=2}^{n}R_{s}(t_{m-2},S^{m-2},E_{s}^{m-2},I_{s}^{m-2},R_{s}^{m-2},E_{r}^{m-2},I_{r}^{m-2},R_{r}^{m-2})\;\times \Pi +\frac{q(△t{)}^{q}}{ABC(q)\Gamma (q+2)}\sum_{m=2}^{n}[R_{s}(t_{m-1},S^{m-1},E_{s}^{m-1},I_{s}^{m-1},R_{s}^{m-1},E_{r}^{m-1},I_{r}^{m-1},\;R_{r}^{m-1})-R_{s}(t_{m-2},S^{m-2},E_{s}^{m-2},I_{s}^{m-2},R_{s}^{m-2},E_{r}^{m-2},I_{r}^{m-2},R_{r}^{m-2})]\times \Theta \;+\frac{q(△t{)}^{q}}{2ABC(q)\Gamma (q+3)}\sum_{m=2}^{n}[R_{s}(t_{m},S^{m},E_{s}^{m},I_{s}^{m},R_{s}^{m},E_{r}^{m},I_{r}^{m},R_{r}^{m})-2R_{s}(t_{m-1},\;S^{m-1},E_{s}^{m-1},I_{s}^{m-1},R_{s}^{m-1},E_{r}^{m-1},I_{r}^{m-1},R_{r}^{m-1})+R_{s}(t_{m-2},S^{m-2},E_{s}^{m-2},I_{s}^{m-2},\;R_{s}^{m-2},E_{r}^{m-2},I_{r}^{m-2},R_{r}^{m-2})]\times \Omega +\frac{q(△t{)}^{q}}{ABC(q)\Gamma (q+1)}\sum_{m=2}^{n}\sigma_{4}{ℋ}_{4}(t_{m-2},R_{s}^{m-2})\;(B_{4}(t_{m-1})-B_{4}(t_{m-2}))\times \Pi +\frac{q(△t{)}^{q}}{ABC(q)\Gamma (q+2)}\sum_{m=2}^{n}[\sigma_{4}{ℋ}_{4}(t_{m-1},E_{s}^{m-1})(B_{4}(t_{m})\;-B_{4}(t_{m-1}))-\sigma_{4}{ℋ}_{4}(t_{m-2},I_{s}^{m-2})(B_{4}(t_{m-1})-B_{4}(t_{m-2}))]\times \Theta \;+\frac{q(△t{)}^{q}}{2ABC(q)\Gamma (q+3)}\sum_{m=2}^{n}[\sigma_{4}{ℋ}_{4}(t_{m},R_{s}^{m})(B_{4}(t_{m+1})-B_{4}(t_{m}))-2\sigma_{4}{ℋ}_{4}(t_{m-1},\;R_{s}^{m-1})(B_{4}(t_{m})-B_{4}(t_{m-1}))+\sigma_{4}{ℋ}_{4}(t_{m-2},R_{s}^{m-2})(B_{4}(t_{m-1})-B_{4}(t_{m-2}))]\times \Omega .$$

$$E_{r}(t_{n+1})=E_{r}(0)+\frac{1-q}{ABC(q)}E_{r}(t_{n},S^{n},E_{s}^{n},I_{s}^{n},R_{s}^{n},E_{r}^{n},I_{r}^{n},R_{r}^{n})\;+\frac{q(△t{)}^{q}}{ABC(q)\Gamma (q+1)}\sum_{m=2}^{n}E_{r}(t_{m-2},S^{m-2},E_{s}^{m-2},I_{s}^{m-2},R_{s}^{m-2},E_{r}^{m-2},I_{r}^{m-2},R_{r}^{m-2})\;\times \Pi +\frac{q(△t{)}^{q}}{ABC(q)\Gamma (q+2)}\sum_{m=2}^{n}[E_{r}(t_{m-1},S^{m-1},E_{s}^{m-1},I_{s}^{m-1},R_{s}^{m-1},E_{r}^{m-1},I_{r}^{m-1},\;R_{r}^{m-1})-E_{r}(t_{m-2},S^{m-2},E_{s}^{m-2},I_{s}^{m-2},R_{s}^{m-2},E_{r}^{m-2},I_{r}^{m-2},R_{r}^{m-2})]\times \Theta \;+\frac{q(△t{)}^{q}}{2ABC(q)\Gamma (q+3)}\sum_{m=2}^{n}[E_{r}(t_{m},S^{m},E_{s}^{m},I_{s}^{m},R_{s}^{m},E_{r}^{m},I_{r}^{m},R_{r}^{m})-2E_{r}(t_{m-1},\;S^{m-1},E_{s}^{m-1},I_{s}^{m-1},R_{s}^{m-1},E_{r}^{m-1},I_{r}^{m-1},R_{r}^{m-1})+E_{r}(t_{m-2},S^{m-2},E_{s}^{m-2},I_{s}^{m-2},\;R_{s}^{m-2},E_{r}^{m-2},I_{r}^{m-2},R_{r}^{m-2})]\times \Omega +\frac{q(△t{)}^{q}}{ABC(q)\Gamma (q+1)}\sum_{m=2}^{n}\sigma_{5}{ℋ}_{5}(t_{m-2},E_{r}^{m-2})\;(B_{5}(t_{m-1})-B_{5}(t_{m-2}))\times \Pi +\frac{q(△t{)}^{q}}{ABC(q)\Gamma (q+2)}\sum_{m=2}^{n}[\sigma_{5}{ℋ}_{5}(t_{m-1},E_{r}^{m-1})\;(B_{5}(t_{m})-B_{5}(t_{m-1}))-\sigma_{5}{ℋ}_{5}(t_{m-2},E_{r}^{m-2})(B_{5}(t_{m-1})-B_{5}(t_{m-2}))]\times \Theta \;+\frac{q(△t{)}^{q}}{2ABC(q)\Gamma (q+3)}\sum_{m=2}^{n}[\sigma_{5}{ℋ}_{5}(t_{m},E_{r}^{m})(B_{5}(t_{m+1})-B_{5}(t_{m}))-2\sigma_{5}{ℋ}_{5}(t_{m-1},\;E_{r}^{m-1})(B_{5}(t_{m})-B_{5}(t_{m-1}))+\sigma_{5}{ℋ}_{5}(t_{m-2},E_{r}^{m-2})(B_{5}(t_{m-1})-B_{5}(t_{m-2}))]\times \Omega .$$

$$I_{r}(t_{n+1})=I_{r}(0)+\frac{1-q}{ABC(q)}I_{r}(t_{n},S^{n},E_{s}^{n},I_{s}^{n},R_{s}^{n},E_{r}^{n},I_{r}^{n},R_{r}^{n})\;+\frac{q(△t{)}^{q}}{ABC(q)\Gamma (q+1)}\sum_{m=2}^{n}I_{r}(t_{m-2},S^{m-2},E_{s}^{m-2},I_{s}^{m-2},R_{s}^{m-2},E_{r}^{m-2},I_{r}^{m-2},R_{r}^{m-2})\;\times \Pi +\frac{q(△t{)}^{q}}{ABC(q)\Gamma (q+2)}\sum_{m=2}^{n}[I_{r}(t_{m-1},S^{m-1},E_{s}^{m-1},I_{s}^{m-1},R_{s}^{m-1},E_{r}^{m-1},I_{r}^{m-1},\;R_{r}^{m-1})-I_{r}(t_{m-2},S^{m-2},E_{s}^{m-2},I_{s}^{m-2},R_{s}^{m-2},E_{r}^{m-2},I_{r}^{m-2},R_{r}^{m-2})]\times \Theta \;+\frac{q(△t{)}^{q}}{2ABC(q)\Gamma (q+3)}\sum_{m=2}^{n}[I_{r}(t_{m},S^{m},E_{s}^{m},I_{s}^{m},R_{s}^{m},E_{r}^{m},I_{r}^{m},R_{r}^{m})-2I_{r}(t_{m-1},\;S^{m-1},E_{s}^{m-1},I_{s}^{m-1},R_{s}^{m-1},E_{r}^{m-1},I_{r}^{m-1},R_{r}^{m-1})+I_{r}(t_{m-2},S^{m-2},E_{s}^{m-2},I_{s}^{m-2},\;R_{s}^{m-2},E_{r}^{m-2},I_{r}^{m-2},R_{r}^{m-2})]\times \Omega +\frac{q(△t{)}^{q}}{ABC(q)\Gamma (q+1)}\sum_{m=2}^{n}\sigma_{6}{ℋ}_{6}(t_{m-2},I_{r}^{m-2})\;(B_{6}(t_{m-1})-B_{6}(t_{m-2}))\times \Pi +\frac{q(△t{)}^{q}}{ABC(q)\Gamma (q+2)}\sum_{m=2}^{n}[\sigma_{6}{ℋ}_{6}(t_{m-1},I_{r}^{m-1})(B_{6}(t_{m})\;-B_{6}(t_{m-1}))-\sigma_{6}{ℋ}_{6}(t_{m-2},I_{r}^{m-2})(B_{6}(t_{m-1})-B_{6}(t_{m-2}))]\times \Theta \;+\frac{q(△t{)}^{q}}{2ABC(q)\Gamma (q+3)}\sum_{m=2}^{n}[\sigma_{6}{ℋ}_{6}(t_{m},I_{r}^{m})(B_{6}(t_{m+1})-B_{6}(t_{m}))-2\sigma_{6}{ℋ}_{6}(t_{m-1},\;I_{r}^{m-1})(B_{6}(t_{m})-B_{6}(t_{m-1}))+\sigma_{6}{ℋ}_{6}(t_{m-2},I_{r}^{m-2})(B_{6}(t_{m-1})-B_{6}(t_{m-2}))]\times \Omega .$$

$$R_{r}(t_{n+1})=R_{r}(0)+\frac{1-q}{ABC(q)}R_{r}(t_{n},S^{n},E_{s}^{n},I_{s}^{n},R_{s}^{n},E_{r}^{n},I_{r}^{n},R_{r}^{n})\;+\frac{q(△t{)}^{q}}{ABC(q)\Gamma (q+1)}\sum_{m=2}^{n}R_{r}(t_{m-2},R^{m-2},E_{s}^{m-2},I_{s}^{m-2},R_{s}^{m-2},E_{r}^{m-2},I_{r}^{m-2},\;R_{r}^{m-2})\times \Pi +\frac{q(△t{)}^{q}}{ABC(q)\Gamma (q+2)}\sum_{m=2}^{n}[R_{r}(t_{m-1},S^{m-1},E_{s}^{m-1},I_{s}^{m-1},R_{s}^{m-1},E_{r}^{m-1},\;I_{r}^{m-1},R_{r}^{m-1}),-R_{r}(t_{m-2},S^{m-2},E_{s}^{m-2},I_{s}^{m-2},R_{s}^{m-2},E_{r}^{m-2},I_{r}^{m-2},R_{r}^{m-2})]\times \Theta \;+\frac{q(△t{)}^{q}}{2ABC(q)\Gamma (q+3)}\sum_{m=2}^{n}[R_{r}(t_{m},S^{m},E_{s}^{m},I_{s}^{m},R_{s}^{m},E_{r}^{m},I_{r}^{m},R_{r}^{m})\;-2R_{r}(t_{m-1},S^{m-1},E_{s}^{m-1},I_{s}^{m-1},R_{s}^{m-1},E_{r}^{m-1},I_{r}^{m-1},R_{r}^{m-1})\;+R_{r}(t_{m-2},S^{m-2},E_{s}^{m-2},I_{s}^{m-2},R_{s}^{m-2},E_{r}^{m-2},I_{r}^{m-2},R_{r}^{m-2})]\times \Omega \;+\frac{q(△t{)}^{q}}{ABC(q)\Gamma (q+1)}\sum_{m=2}^{n}\sigma_{7}{ℋ}_{7}(t_{m-2},R_{r}^{m-2})(B_{7}(t_{m-1})-B_{7}(t_{m-2}))\times \Pi \;+\frac{q(△t{)}^{q}}{ABC(q)\Gamma (q+2)}\sum_{m=2}^{n}[\sigma_{7}{ℋ}_{7}(t_{m-1},R_{r}^{m-1})(B_{7}(t_{m})-B_{7}(t_{m-1}))\;-\sigma_{7}{ℋ}_{7}(t_{m-2},R_{r}^{m-2})(B_{7}(t_{m-1})-B_{7}(t_{m-2}))]\times \Theta \;+\frac{q(△t{)}^{q}}{2ABC(q)\Gamma (q+3)}\sum_{m=2}^{n}[\sigma_{7}{ℋ}_{7}(t_{m},R_{r}^{m})(B_{7}(t_{m+1})-B_{7}(t_{m}))-2\sigma_{7}{ℋ}_{7}(t_{m-1},\;R_{r}^{m-1})(B_{7}(t_{m})-B_{7}(t_{m-1}))+\sigma_{7}{ℋ}_{7}(t_{m-2},R_{r}^{m-2})(B_{7}(t_{m-1})-B_{7}(t_{m-2}))]\times \Omega .$$

Where,

$\Pi =(n-m+1{)}^{q}-(n-m{)}^{q}$,

$\Theta =(n-m+1{)}^{q}(2q+3+n-m)-(n-m{)}^{q}(3q+3+n-m)$,

$\Omega =(n-m+1{)}^{q}[2(n-m{)}^{2}+(3q+10)(n-m)+2q^{2}+9q+12]-(n-m{)}^{q}[2(k-m{)}^{2}+(5q+10)(n-m)+6q^{2}+18q+12]$.

## Numerical results and discussions

### 1. Predictions of different interventions

To formulate more effective interventions for attaining the End TB strategy, this section predicts the impact of the four single measures along with their combinations on the count of individuals afflicted with DR-TB.

#### 1.1 Adoption of single measures.

**Fig 3:**
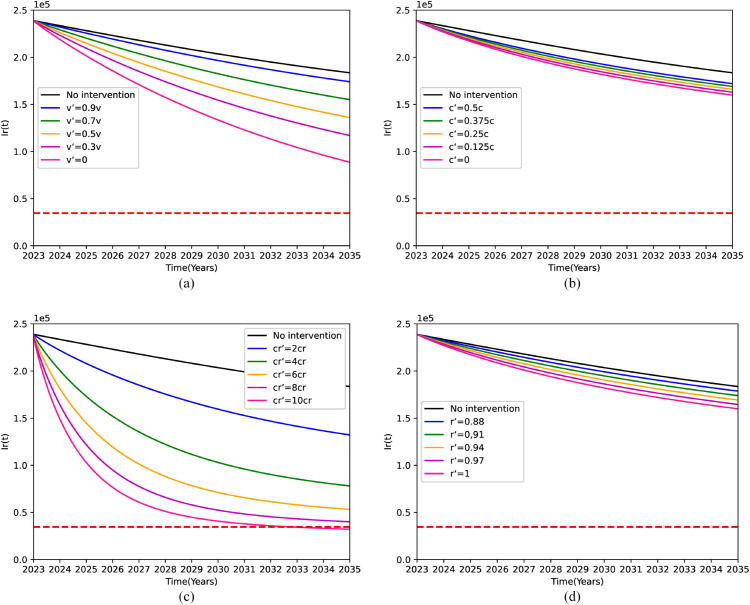
Projected number of drug-resistant tuberculosis (DR-TB) patients in 2035 under different single-intervention strategies. (a) Reducing the v. (b) Improving the *c*. (c) Reducing the *c*_*r*_. (d) Improving the r.

Fig 3 illustrates the effect on the number of patients with DR-TB when each of the four single measures is implemented separately:

We can get trends in the number of patients with DR-TB by screening for latent DR-TB infection *E*_*r*_ and thereby reducing the rate of disease progression (*v*) in patients with *E*_*r*_, this intervention decreased v by 10%, 30%, 60%, 90%, and 100% compared to the initial value. The simulation results in Fig 3(a) indicate that at the same time point, *v* is significantly negatively correlated with the number of *I*_*r*_, meaning that the lower the disease progression rate, the fewer the number of patients. However, even when *v* is reduced to zero, the number of patients with DR-TB cannot fall below the red dotted line by 2035. That is, even if all *E*_*r*_ do not turn into patients with *I*_*r*_, the target will not be met by 2035.

By increasing the awareness and standard of treatment for DS-TB patients, which in turn reduces the conversion rate (*c*) from DS-TB to DR-TB patients, the intervention reduces *c* by 50%, 62.5%, 75%, 87.5%, and 100% relative to the current 0.08. The graph in Fig 3(b) demonstrates that concurrently, as *c* diminishes, there is a corresponding decrease in the number of DR-TB patients. However, alternatively, despite the conversion rate dropping to 0, the DR-TB case count will still remain above the 90% reduction target by the year 2035.

Fig 3(c) illustrates that there exists an inverse relationship between the number of DR-TB patients(*I*_*r*_) and the cure rate of DR-TB (*c*_*r*_) patients. That is, as the cure rate of DR-TB patients steadily increases, the number of DR-TB patients concomitantly decreases. Given that the current cure rate stands at 0.0493, if it continuously rises and reaches 10 times the current rate (10*c*_*r*_) by the year 2035, it can be projected that the number of DR-TB patients will witness a significant reduction of 314,097 cases. This reduction accounts for a substantial 90.72% when compared to the level observed in 2015. Consequently, it can be inferred that by substantially enhancing the cure rate of DR-TB patients, the targeted goal can be attained by the year 2035.

Fig 3(d) shows that increasing the proportion of DS-TB cured cases (*r*) has a positive effect on the reduction of DR-TB patients. Specifically, when *r* is gradually increased from 0.85 to 0.88, 0.91, 0.94, 0.97, and ultimately reaches 1, it can be observed that the proportion of cured cases among DS-TB patients ascends to 100%. Nevertheless, it is projected that by the year 2035, the number of DR-TB patients will only experience a reduction of 186,343 cases. In relative terms, this represents a 53.8% decrease when compared to the level witnessed in 2015. This reduction fails to meet the target of achieving a 90% reduction in the number of cases.

#### 1.2 Taking joint measures.

**Fig 4:**
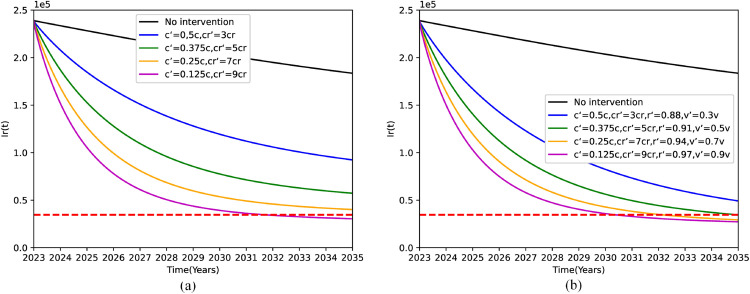
Projected number of drug-resistant tuberculosis (DR-TB) patients in 2035 under different joint measures strategies. (a) Decreasing the c while increasing the *c*_*r*_. (b) Increasing the r and *c*_*r*_, reducing the v and c.

Fig 4 illustrates the impact on the number of patients with DR-TB when joint measures are taken:

By enhancing the efficacy and the standard of care for DR-TB patients, we aim to reduce the conversion rate of DS-TB into DR-TB cases(*c*), while concurrently enhancing the cure rate of DR-TB patients (*c*_*r*_). Fig 4(a) indicates that when *c* decreases to 0.01 and *c*_*r*_ increases to 0.45 simultaneously, it can be projected that by the year 2035, the number of DR-TB patients will experience a reduction of 315,773 cases. In relative terms, this represents a 91.2% decrease compared to the level observed in 2015. However, implementing either a reduction in *c* to 0.01 alone or an increase in *c*_*r*_ to 0.45 alone will prove insufficient to attain the predefined goal. Consequently, it is evident that the combined implementation of these measures exhibits a superior effect in contrast to the application of individual measures.

Fig 4(b) demonstrates the impact of the intervention of the four jointed measures on the number of patients with DR-TB. By simultaneously implementing strategies to decrease the conversion rate of DS-TB to DR-TB patients (*c*), enhance the cure rate of DR-TB patients (*c*_*r*_), elevate the proportion of cured DS-TB cases (*r*), and diminish the disease progression rate (*v*) of DR-TB, significant outcomes can be observed. it has been found that when *c* is maintained at 0.3, *c*_*r*_ attains a value of 0.25, *r* exceeds 91%, and *v* is contained at 3.55*e*^−5^, the population of DR-TB patients will witness a decline of 311,641 cases by the year 2035. This remarkable reduction accounts for 90.01% when compared to the baseline level in 2015, thereby effectively fulfilling the objective of achieving a 90% decrease in DR-TB cases by 2035. It is noteworthy that should the combined measures be further intensified, there exists the possibility of meeting the target ahead of the scheduled time.

### 2. Relationship between the number of persons in each category and the order q

In this section, we perform numerical simulation of the fractional stochastic differential equations model. We conduct numerical simulations for different fractional orders model by the Atangana-Baleanu-Caputo differential operator and the numerical method of Newton polynomial interpolation.

**Fig 5:**
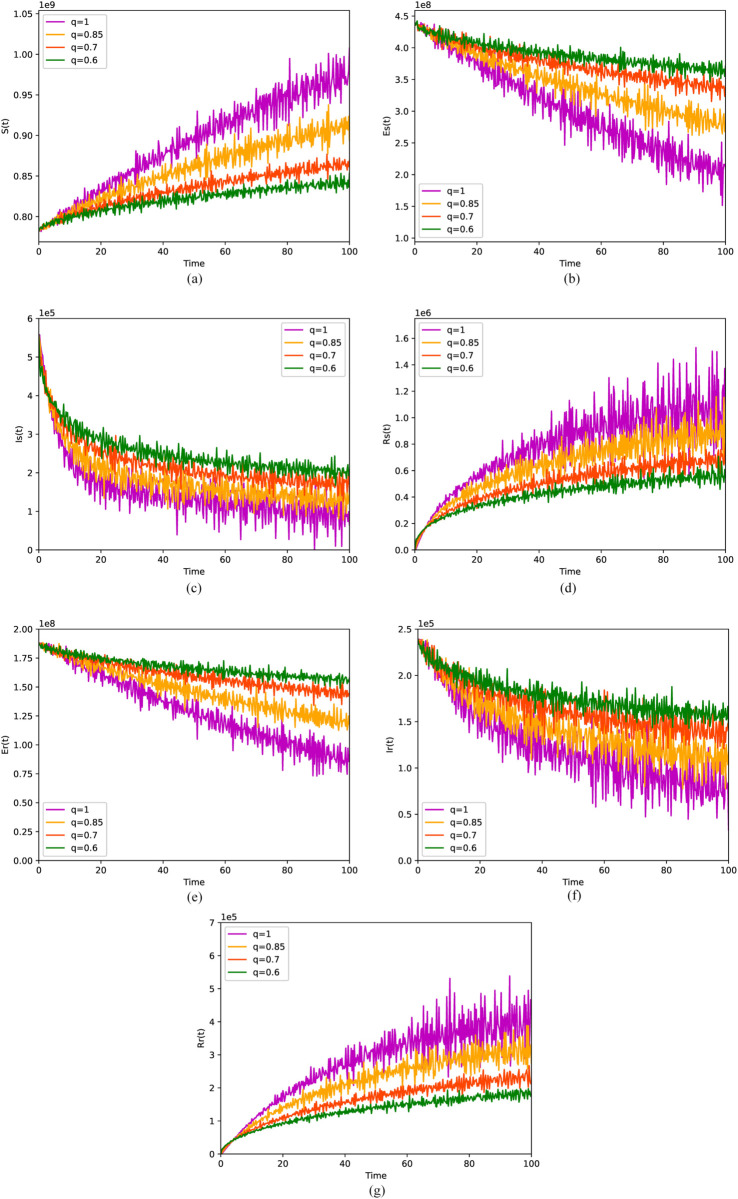
Solutions of the model (6) with ABC operator for four values of the order q when. $\sigma_{1}=0.003$, $\sigma_{2}=0.010$, $\sigma_{3}=0.030$, $\sigma_{4}=0.040$, $\sigma_{5}=0.010$, $\sigma_{6}=0.025$, $\sigma_{7}=0.035.$ (a) Stochastic behavior of *S*. (b) Stochastic behavior of *E*_*s*_. (c) Stochastic behavior of *I*_*s*_. (d) Stochastic behavior of *R*_*s*_. (e) Stochastic behavior of *E*_*r*_. (f) Stochastic behavior of *I*_*r*_.

**Fig 6:**
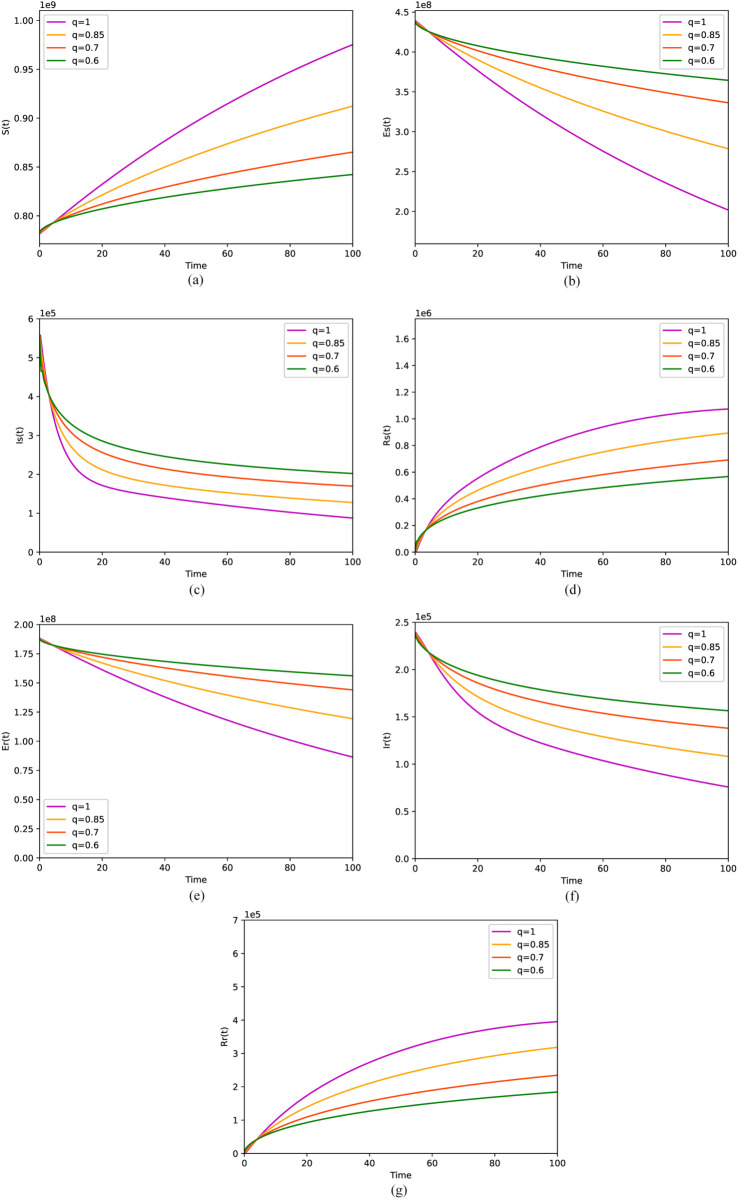
Solutions of the model (6) with ABC operator for four values of the order q. (a) Deterministic behavior of *S*. (b) Deterministic behavior of *E*_*s*_. (c) Deterministic behavior of *I*_*s*_. (d) Deterministic behavior of *R*_*s*_. (e) Deterministic behavior of *E*_*r*_. (f) Deterministic behavior of *I*_*r*_. (g) Deterministic behavior of *R*_*r*_.

Figs 5 and 6 respectively describe the numerical simulation results of the solutions derived from the fractional stochastic differential equation model (6) and the fractional differential equation model (1), at varying orders of *q*, under the condition where *R*_0_ is less than 1. Figs 5(a), 5(d), 5(g) and 6(a), 6(d), 6(g) both show that the number of susceptible, DS-TB and DR-TB recovered individuals gradually increases with increasing order *q*. Meanwhile, Figs 5(b), 5(c), 5(e), 5(f) and 6(b), 6(c), 6(e), 6(f) depict that the number of individuals infected with DS-TB and DR-TB, as well as the number of individuals latently infected with DS-TB and DR-TB, both decrease with the increasing order *q*. That is to say, although the fluctuations are observed in the solution of model (1) due to the incorporation of the random term, the overall trend remains consistent with that observed in the absence of the random term. Through numerical simulations, it has been discerned that the memory effect inherent in the disease’s spreading process exerts a significant influence on its dissemination, which is not adequately captured by an integer-order model.

### 3. Impact of stochastic disturbances on disease transmission

**Fig 7:**
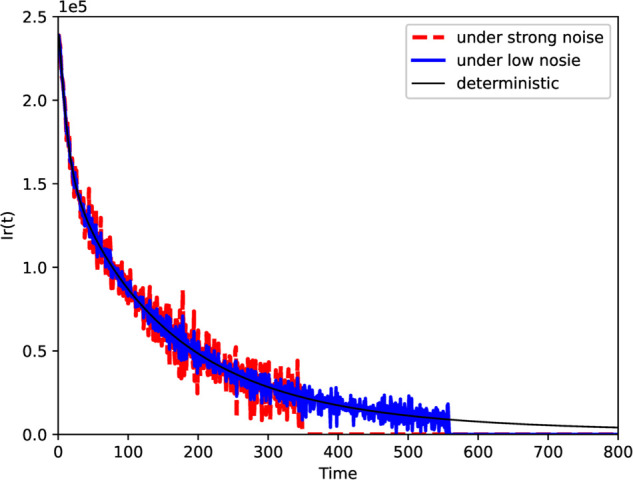
The solution curves of model for different noise intensities.

In this section, we investigate the effect of noise intensity on the number of patients with DR-TB. Select q=0.95 and the strong noise parameters: $\sigma_{1}=0.0006$, $\sigma_{2}=0.0016$, $\sigma_{3}=0.0040$, $\sigma_{4}=0.0020$, $\sigma_{5}=0.0020$, $\sigma_{6}=0.0030$, $\sigma_{7}=0.0024$ and the low noise parameters: $\sigma_{1}=0.0003$, $\sigma_{2}=0.0008$, $\sigma_{3}=0.0020$, $\sigma_{4}=0.0010$, $\sigma_{5}=0.0010$, $\sigma_{6}=0.0015$, $\sigma_{7}=0.0012$. As shown in Fig 7, the solution of the fractional stochastic model is perturbed around the curve of the solution of the fractional order deterministic model, with the degree of perturbation related to the intensity of the noise. By comparison, it is found that when the noise intensity is relatively low, the effect of random interference on the stochastic model is weak. However, high-intensity noise can rapidly reduce the number of infected individuals, thus effectively controlling the spread of DR-TB.

## Conclusion

In this paper, we incorporate stochasticity in disease transmission within a fractional order model based on Atangana-Baleanu-Caputo operator to analyze its effect on DR-TB transmission. The non-negative boundedness of the solutions of is investigated and the basic reproduction number is calculated. Using real data on DR-TB patients in China from 2008 to 2023, we employed the least squares method for fitting. After experimenting with various parameter values, we found that the model achieved better fitting results when *q* was set to 0.95. Based on this, we have predicted the number of DR-TB patients from 2024 to 2035, and it is anticipated that without implementing control strategies, it will be challenging to achieve the set targets by 2035. The existence and uniqueness of solutions for the DR-TB model under stochastic conditions was subsequently proved theoretically and the Atangana-Baleanu-Caputo fractional stochastic model was solved numerically using Newtonian polynomial interpolation techniques.

We predicted the number of DR-TB patients in 2035 by adopting four single intervention measures and joint measures respectively. After comparison, we found that the effect of joint measures was better than that of single measures. Furthermore, numerical simulations were conducted to plot the time evolution of solutions for both of fractional stochastic models and deterministic models with different orders. It can be found that both the memory effect and the random effect will affect the disease transmission, and the disease development trends of the models with and without random terms are consistent. To investigate the impact of random disturbance intensity on disease transmission, we compared strong noise with low noise and found that the degree of disturbance of the solution of the fractional stochastic model is related to the noise intensity. The greater the noise, the greater the degree of disturbance, and higher noise may lead to the early disappearance of DR-TB.

Therefore, through analysis, we can conclude that the strategy can be achieved by 2035 by vigorously increasing the cure rate of DR-TB patients or simultaneously controlling the DS-TB to DR-TB conversion rate at 0.3, the cure rate of DR-TB at 0.25, the proportion of DS-TB cured cases at 91%, and the progression rate of disease at $3.55\times 10^{-5}$. On the other hand, even if DR-TB persists in the deterministic model, when the environment is subject to strong random disturbances, it is also possible to achieve the End TB Strategy by 2035.

## References

[1] World Health Organization. ea. type. 2023. https://www.who.int/teams/global-tuberculosis-programme/tb-reports

[2] Dadu A, Hovhannesyan A, Ahmedov S, van der Werf MJ, Dara M. Drug-resistant tuberculosis in eastern Europe and central Asia: a time-series analysis of routine surveillance data. Lancet Infect Dis. 2020;20(2):250–8. doi: 10.1016/S1473-3099(19)30568-7 31784371

[3] Waaler H, Geser A, Andersen S. The use of mathematical models in the study of the epidemiology of tuberculosis. Am J Public Health Nations Health. 1962;52(6):1002–13. doi: 10.2105/ajph.52.6.1002 14004185 PMC1523050

[4] Ullah I, Ahmad S, Zahri M. Investigation of the effect of awareness and treatment on Tuberculosis infection via a novel epidemic model. Alexandria Engineering Journal. 2023;68:127–39. doi: 10.1016/j.aej.2022.12.061

[5] Yu Y, Shi Y, Yao W. Dynamic model of tuberculosis considering multi-drug resistance and their applications. Infect Dis Model. 2018;3:362–72. doi: 10.1016/j.idm.2018.11.001 30839915 PMC6326219

[6] Yusuf TT, Abidemi A. Effective strategies towards eradicating the tuberculosis epidemic: an optimal control theory alternative. Healthcare Analytics. 2023;3:100131. doi: 10.1016/j.health.2022.100131

[7] Ronoh M, Jaroudi R, Fotso P, Kamdoum V, Matendechere N, Wairimu J, et al. A mathematical model of tuberculosis with drug resistance effects. AM. 2016;07(12):1303–16. doi: 10.4236/am.2016.712115

[8] Atangana A, Jain S. Models of fluid flowing in non-conventional media: new numerical analysis. Discrete & Continuous Dynamical Systems - S. 2020;13(3):467–84. doi: 10.3934/dcdss.2020026

[9] Yavuz M, Akman M, Usta F, Özdemir N. Effect of the awareness parameter on a fractional-order tuberculosis model. In: AIP Conference Proceedings. 2022. 070006. 10.1063/5.0114888

[10] Evirgen F. Transmission of Nipah virus dynamics under Caputo fractional derivative. Journal of Computational and Applied Mathematics. 2023;418:114654. doi: 10.1016/j.cam.2022.114654

[11] Baleanu D, Qureshi S, Yusuf A, Soomro A, Osman MS. Bi-modal COVID-19 transmission with Caputo fractional derivative using statistical epidemic cases. Partial Differential Equations in Applied Mathematics. 2024;10:100732. doi: 10.1016/j.padiff.2024.100732

[12] Kubra KT, Gulshan S, Ali R. An Atangana–Baleanu derivative-based fractal-fractional order model for the monkey pox virus: a case study of USA. Partial Differential Equations in Applied Mathematics. 2024;9:100623. doi: 10.1016/j.padiff.2024.100623

[13] Rahman MU, Arfan M, Shah Z, Kumam P, Shutaywi M. Nonlinear fractional mathematical model of tuberculosis (TB) disease with incomplete treatment under Atangana-Baleanu derivative. Alexandria Engineering Journal. 2021;60(3):2845–56. doi: 10.1016/j.aej.2021.01.015

[14] Omame A, Abbas M, Onyenegecha CP. A fractional-order model for COVID-19 and tuberculosis co-infection using Atangana-Baleanu derivative. Chaos Solitons Fractals. 2021;153:111486. doi: 10.1016/j.chaos.2021.111486 34658543 PMC8501266

[15] Padder A, Almutairi L, Qureshi S, Soomro A, Afroz A, Hincal E, et al. Dynamical analysis of generalized tumor model with caputo fractional-order derivative. Fractal Fract. 2023;7(3):258. doi: 10.3390/fractalfract7030258

[16] Din A. The stochastic bifurcation analysis and stochastic delayed optimal control for epidemic model with general incidence function. Chaos. 2021;31(12):123101. doi: 10.1063/5.0063050 34972335

[17] Din A, Li Y, Yusuf A. Delayed hepatitis B epidemic model with stochastic analysis. Chaos, Solitons & Fractals. 2021;146:110839. doi: 10.1016/j.chaos.2021.110839

[18] Ali Khan W, Zarin R, Zeb A, Khan Y, Khan A. Navigating food allergy dynamics via a novel fractional mathematical model for antacid-induced allergies. JMTM. 2024;1(1):25–51. doi: 10.56868/jmtm.v1i1.3

[19] tul Ain Q. Nonlinear stochastic cholera epidemic model under the influence of noise. Journal of Mathematical Techniques in Modeling. 2024;2024(1):52–74. doi: 10.56868/jmtm.v1i1.30

[20] Uçar S, Koca I, Özdemir N, İnci T. A stochastic approach to tumor modeling incorporating macrophages. BBM. 2024. doi: 10.59292/bulletinbiomath.2024007

[21] Andrawus J, Ahmad YU, Andrew AA, Yusuf A, Qureshi S, Denue BA, et al. Impact of surveillance in human-to-human transmission of monkeypox virus. Eur Phys J Spec Top. 2024;234(3):483–514. doi: 10.1140/epjs/s11734-024-01346-5

[22] Naik PA, Yeolekar BM, Qureshi S, Yeolekar M, Madzvamuse A. Modeling and analysis of the fractional-order epidemic model to investigate mutual influence in HIV/HCV co-infection. Nonlinear Dyn. 2024;112(13):11679–710. doi: 10.1007/s11071-024-09653-1

[23] Arora S, Suman HK, Mathur T, Pandey HM, Tiwari K. Fractional derivative based weighted skip connections for satellite image road segmentation. Neural Netw. 2023;161:142–53. doi: 10.1016/j.neunet.2023.01.031 36745939

[24] Alkahtani BST, Alzaid SS. Stochastic mathematical model of Chikungunya spread with the global derivative. Results in Physics. 2021;20:103680. doi: 10.1016/j.rinp.2020.103680

[25] Bonyah E, Yuan Y, Mangal S. Fractional stochastic modelling of dengue fever: the social awareness perspective. Scientific African. 2023;22:e01966. doi: 10.1016/j.sciaf.2023.e01966

[26] Ul Abadin Zafar Z, DarAssi MH, Ahmad I, Assiri TA, Meetei MZ, Khan MA, et al. Numerical simulation and analysis of the stochastic HIV/AIDS model in fractional order. Results in Physics. 2023;53:106995. doi: 10.1016/j.rinp.2023.106995

[27] Atangana A, Araz SI. Fractional stochastic differential equations: applications to Covid-19 modeling. Springer Nature; 2022.

[28] Bonyah E, Panigoro HS, Fatmawati, Rahmi E, Juga ML. Fractional stochastic modelling of monkeypox dynamics. Results in Control and Optimization. 2023;12:100277. doi: 10.1016/j.rico.2023.100277

[29] Chukwu CW, Bonyah E, Juga ML, Fatmawati. On mathematical modeling of fractional-order stochastic for tuberculosis transmission dynamics. Results in Control and Optimization. 2023;11:100238. doi: 10.1016/j.rico.2023.100238

[30] Mangal S, Bonyah E, Sharma VS, Yuan Y. A novel fractional-order stochastic epidemic model to analyze the role of media awareness in the spread of conjunctivitis. Healthcare Analytics. 2024;5:100302. doi: 10.1016/j.health.2024.100302

[31] Rashid S, Jarad F. Novel investigation of stochastic fractional differential equations measles model via the white noise and global derivative operator depending on Mittag-Leffler Kernel. CMES. 2024;139(3):2289–327. doi: 10.32604/cmes.2023.028773

[32] World Health Organization. ea type. 2014. https://www.who.int/teams/global-tuberculosis-programme/tb-reports

[33] Atangana A, Baleanu D. New fractional derivatives with nonlocal and non-singular kernel: theory and application to heat transfer model. arXiv preprint 2016. doi: arXiv:160203408

[34] Din A, Li Y, Khan FM, Khan ZU, Liu P. On analysis of fractional order mathematical model of hepatitis b using atangana–baleanu caputo (ABC) derivative. Fractals. 2021;30(01):2240017.doi: 10.1142/s0218348x22400175

[35] Xu A, Wen Z-X, Wang Y, Wang W-B. Prediction of different interventions on the burden of drug-resistant tuberculosis in China: a dynamic modelling study. J Glob Antimicrob Resist. 2022;29:323–30. doi: 10.1016/j.jgar.2022.03.018 35351676

[36] van den Driessche P, Watmough J. Reproduction numbers and sub-threshold endemic equilibria for compartmental models of disease transmission. Math Biosci. 2002;180:29–48. doi: 10.1016/s0025-5564(02)00108-6 12387915

[37] People’s Republic of China NHC. Type. http://www.nhc.gov.cn/jkj/s2907/new_list.shtml

[38] Type. http://data.stats.gov.cn/tablequery.htm?code=AD03

[39] Atangana A, Araz SI. New numerical scheme with Newton polynomial: theory, methods, and applications. Academic Press; 2021.

